# Hippocampal Sharp-Wave Ripples Influence Selective Activation of the Default Mode Network

**DOI:** 10.1016/j.cub.2016.01.017

**Published:** 2016-03-07

**Authors:** Raphael Kaplan, Mohit H. Adhikari, Rikkert Hindriks, Dante Mantini, Yusuke Murayama, Nikos K. Logothetis, Gustavo Deco

**Affiliations:** 1 Wellcome Trust Centre for Neuroimaging, UCL Institute of Neurology, University College London, 12 Queen Square, London WC1N 3BG, UK; 2 Center for Brain and Cognition, Departament de Tecnologies de la Informació I les Comunicacions, Universitat Pompeu Fabra, Roc Boronat 138, 08018 Barcelona, Spain; 3 Department of Health Sciences and Technology, ETH Zurich, 8057 Zurich, Switzerland; 4 Movement Control and Neuroplasticity Research Group, KU Leuven, 3001 Leuven, Belgium; 5 Max Planck Institute for Biological Cybernetics, 72076 Tübingen, Germany; 6 Imaging Science and Biomedical Engineering, University of Manchester, Manchester M13 9PT, UK; 7 Institució Catalana de la Recerca i Estudis Avançats (ICREA), Universitat Pompeu Fabra, Passeig Lluís Companys 23, Barcelona 08010, Spain

## Abstract

The default mode network (DMN) is a commonly observed resting-state network (RSN) that includes medial temporal, parietal, and prefrontal regions involved in episodic memory [1, 2, 3]. The behavioral relevance of endogenous DMN activity remains elusive, despite an emerging literature correlating resting fMRI fluctuations with memory performance [4, 5]—particularly in DMN regions [6, 7, 8]. Mechanistic support for the DMN’s role in memory consolidation might come from investigation of large deflections (sharp-waves) in the hippocampal local field potential that co-occur with high-frequency (>80 Hz) oscillations called ripples—both during sleep [9, 10] and awake deliberative periods [11, 12, 13]. Ripples are ideally suited for memory consolidation [14, 15], since the reactivation of hippocampal place cell ensembles occurs during ripples [16, 17, 18, 19]. Moreover, the number of ripples after learning predicts subsequent memory performance in rodents [20, 21, 22] and humans [23], whereas electrical stimulation of the hippocampus after learning interferes with memory consolidation [24, 25, 26]. A recent study in macaques showed diffuse fMRI neocortical activation and subcortical deactivation specifically after ripples [27]. Yet it is unclear whether ripples and other hippocampal neural events influence endogenous fluctuations in specific RSNs—like the DMN—unitarily. Here, we examine fMRI datasets from anesthetized monkeys with simultaneous hippocampal electrophysiology recordings, where we observe a dramatic increase in the DMN fMRI signal following ripples, but not following other hippocampal electrophysiological events. Crucially, we find increases in ongoing DMN activity after ripples, but not in other RSNs. Our results relate endogenous DMN fluctuations to hippocampal ripples, thereby linking network-level resting fMRI fluctuations with behaviorally relevant circuit-level neural dynamics.

## Highlights

• Behavioral relevance of offline fluctuations in the default mode network is unclear
• Hippocampal sharp-wave ripples during rest are linked with memory consolidation
• Default mode network signal increases after ripples, but not other hippocampal events
• Other neocortical resting-state networks do not show similar changes after ripples

## Teaser

Kaplan et al. show that offline fMRI signal fluctuations throughout the default mode network (DMN), which includes neocortical regions important for episodic memory, are uniformly influenced by hippocampal sharp-wave ripples. These results relate ongoing DMN fluctuations to the consolidation of past experience and preparation for future behavior.

## Results

We present novel analyses conducted on fMRI datasets from two anesthetized macaques used in a prior study by Logothetis and colleagues [27], where we ascertained whether there were changes at the level of whole-brain resting-state networks (RSNs) after hippocampal hpsigma (8–22 Hz), gamma (25–75 Hz), or ripple (80–180 Hz) events. We implemented a recently developed technique that uses spatial independent component analysis (ICA) to define correlated fMRI signal fluctuations measured across multiple scan experiments/sessions and subjects into component brain networks [28, 29]. Analyzing 25 fMRI sessions each lasting 10 min in both subjects, we isolated the macaque equivalent of the default mode network (DMN) and compared it to the most robustly observed RSN across sessions and monkeys in our data, the ventral somatomotor network. First, we investigated whether there were positive DMN blood-oxygen-level-dependent (BOLD) signal responses after hippocampal ripples and whether these responses also occurred after the onset of hippocampal hpsigma and gamma events. Second, we investigated whether these three different hippocampal events also co-occurred with BOLD signal fluctuations in the ventral somatomotor network, a RSN not implicated in hippocampal-dependent memory consolidation. Consequently, we could determine whether RSN responses were network- and neural-event specific.

**Figure 1:**
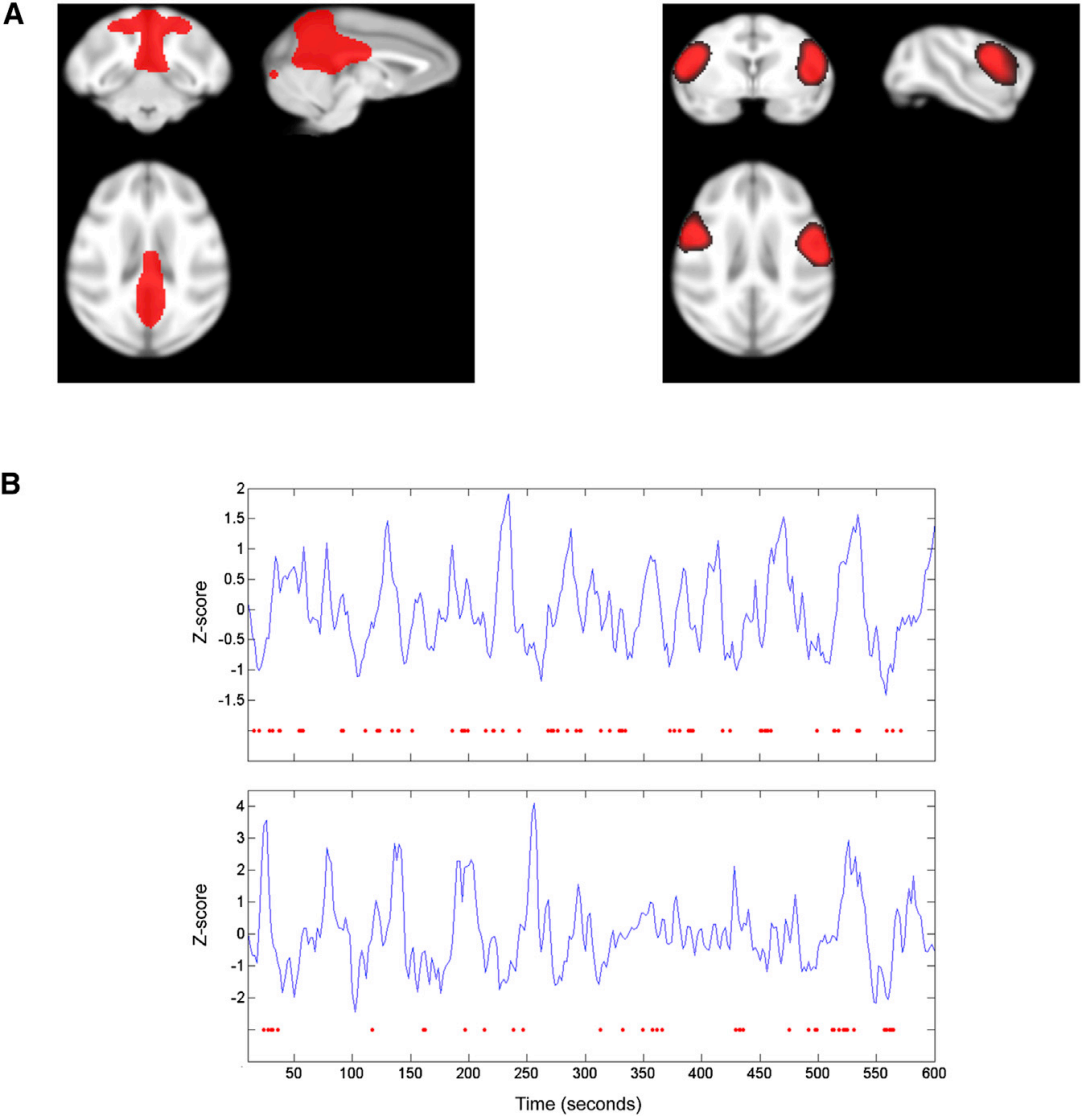
Default Mode and Ventral Somatomotor Resting-State Networks (A) Group-level fixed-effects image of default mode network (DMN; left) and ventral somatomotor network (VSN; right) in two rhesus monkeys. Networks are shown at slices most representative of the correlation pattern on which network identification was based. Images were statistically thresholded at Z > 2 and overlaid on a composite structural from the UWRMAC-DTI271 atlas space. See Figure S1 for other resting-state independent components (ICs) present in both monkeys. (B) DMN time course for a representative 10 min session in monkey 1 (top plot) and monkey 2 (bottom plot), where red dots below represent the onset of hippocampal ripple events. Representative sessions were chosen based on closeness to mean IC rank out of all present ICs for a given session (monkey 1: mean = 4.04, displayed = 3; monkey 2: mean = 6.64, displayed = 7) and ripple amount (monkey 1: mean = 68.8, displayed = 69; monkey 2: mean = 41.2, displayed = 39).

We first used spatial ICA on fMRI experiments/sessions from two monkeys to define RSNs of brain areas showing correlated fMRI activity. After performing a cluster analysis to establish the topological correspondence of RSNs across sessions and monkeys, we isolated the DMN and ventral somatomotor network (Figure 1; see Figure S1 for remaining RSNs). fMRI independent component (IC) time courses for the DMN and ventral somatomotor network were then aligned to the onset of hpsigma (monkey 1: n_events_ = 1117; monkey 2: n_events_ = 887), gamma (monkey 1: n_events_ = 823; monkey 2: n_events_ = 917), and ripple (monkey 1: n_events_ = 1720; monkey 2: n_events_ = 911) events, and their averages were convolved with a hemodynamic response function (HRF). These average event-related BOLD signals were subsequently used as regressors in a standard event-related fMRI design. We report statistics for each monkey across sessions. To determine whether the HRF accurately captured the BOLD response to each event, we also plotted the evoked BOLD response for both RSNs without any fitting.

### Effect of Neural Events on RSNs

#### Monkey 1

**Figure 2:**
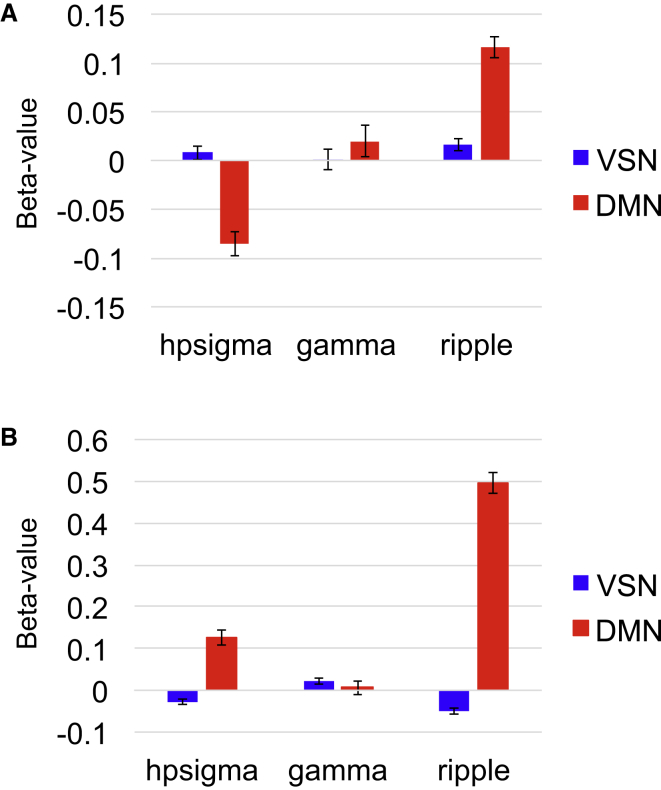
Influence of Hippocampal Neural Events on DMN and VSN (A) Beta values for each network and neural event for monkey 1 (mean ± SEM). There were significantly higher DMN activations after ripples compared to hpsigma (t(24) = 5.99, p < 0.001) or gamma (t(24) = 2.50, p = 0.020) events. Additionally, there was significantly higher DMN versus VSN activity (t(24) = 3.62, p = 0.001) after ripples. There was also a significant decrease in DMN activity (t(24) = −3.55, p = 0.002) after hpsigma events, also when compared to gamma events (t(24) = −2.45, p = 0.022). DMN activity was also significantly lower (t(24) = −3.05, p = 0.006) compared to VSN activity after hpsigma events. (B) Beta values for each network and neural event for monkey 2 (mean ± SEM). There was significantly higher DMN activity after ripples compared to hpsigma (t(21) = 6.48, p < 0.001) or gamma (t(20) = 7.42, p < 0.001) events. Additionally, there was significantly higher DMN versus VSN activity after ripples (t(21) = 11.6, p < 0.001). Converse to monkey 1, there was a significant increase in DMN activity after hpsigma events (t(21) = 3.25, p = 0.004), which was significantly higher (t(20) = 2.26, p = 0.035) than after DMN gamma events and also higher (t(21) = 3.86, p = 0.001) than VSN activity after hpsigma events. There was a significant decrease in ventral somatomotor activity after the onset of ripple events (t(24) = −3.06, p = 0.006). See Figure S2 for mean plots averaged across both monkeys, along with plots showing effect of ripples on other neocortical RSNs.

Using a 2 × 3 within-session repeated-measures ANOVA for network by neural event, we found a significant interaction (F(2,23) = 11.9, p < 0.001; Figure 2A). We also found a main effect for neural event (F(2,23) = 14.9, p < 0.001) but not network (F(1,24) = 0.210, p = 0.651). This interaction was driven by positive DMN BOLD responses after ripples (t(24) = 5.26, p < 0.001). Paired t tests revealed that there were significantly higher DMN activations after ripples compared to hpsigma (t(24) = 5.99, p < 0.001) or gamma (t(24) = 2.50, p = 0.020) events. Additionally, there was significantly higher DMN versus ventral somatomotor network activity (t(24) = 3.62, p = 0.001) after ripples.

For the other neural events, we found a significant decrease in DMN activity (t(24) = −3.55, p = 0.002) after hpsigma events. DMN activity after gamma events was significantly lower for hpsigma versus gamma events (t(24) = −2.45, p = 0.022). DMN activity was also significantly lower (t(24) = −3.05, p = 0.006) compared to ventral somatomotor activity after hpsigma events. Otherwise, we observed no significant differences between events for ventral somatomotor network activity. Furthermore, there were no other significant changes versus baseline in either network for the other neural events. See Table 1 for a complete listing of one-sample t values for DMN and ventral somatomotor network activity after hpsigma, gamma, and ripple events and Table S1 for DMN correlations with left and right hippocampus after ripples.

#### Monkey 2

Using a 2 × 3 within-session repeated-measures ANOVA for network by neural event, we found a significant interaction (F(2,18) = 36.2, p < 0.001; Figure 2B). We also found a main effect for both neural event (F(2,18) = 14.3, p < 0.001) and network (F(1,19) = 59.1, p < 0.001). Once again, this effect was related to DMN increases after ripple events (t(21) = 9.41, p < 0.001). In follow-up paired t tests, there was significantly higher DMN activity after ripples compared to hpsigma (t(21) = 6.48, p < 0.001) or gamma (t(20) = 7.42, p < 0.001) events. Additionally, there was significantly higher DMN versus ventral somatomotor network activity after ripples (t(21) = 11.6, p < 0.001).

Converse to monkey 1, there was a significant increase in DMN activity after hpsigma events (t(21) = 3.25, p = 0.004). DMN activity after hpsigma events was significantly higher (t(20) = 2.26, p = 0.035) than after gamma events and higher (t(21) = 3.86, p = 0.001) than ventral somatomotor activity after hpsigma events. Additionally, there was a significant decrease in ventral somatomotor activity after the onset of ripple events (t(24) = −3.06, p = 0.006), but not after any other events (see Table 1 for a complete listing of t values for both the response of both networks to each event and Table S1 for DMN correlations with left and right hippocampus after ripples). Lastly, there was significantly lower ventral somatomotor network activity after ripples than after gamma events (t(23) = 3.04, p = 0.006), while there was a trend (t(23) = 2.07, p = 0.050) for higher ventral somatomotor activity for gamma versus hpsigma events. See Figure S2A of the aforementioned effects averaged across both monkeys. To add further specificity to our findings, we investigated three other neocortical RSNs with ICs that were less robust than the DMN and VSN but still observable in more than half of the datasets in both monkeys: the primary visual, occipitotemporal, and frontoparietal networks (Figure S2B). Notably, we observed only negligible BOLD changes after ripples in these three RSNs across both monkeys (Figure S2B).

### Temporal Profile of Effect of Neural Events on RSNs

**Figure 3:**
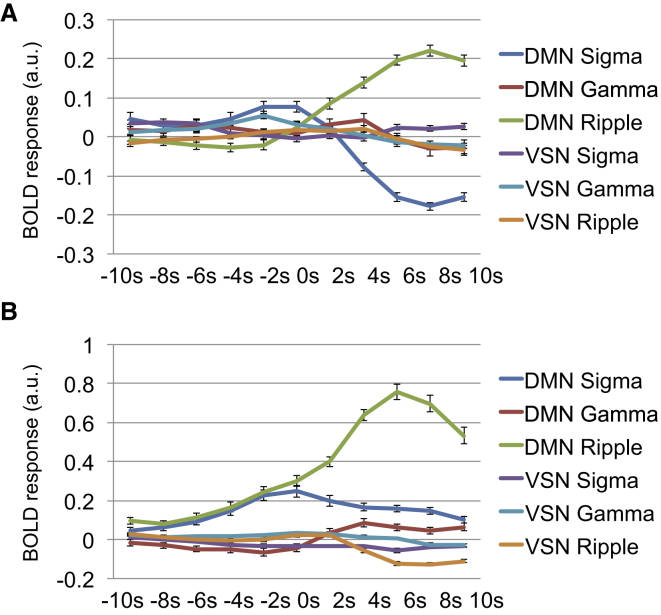
Time Course of Hippocampal Neural Events in DMN and VSN (A) Evoked response values (signal amplitude presented in arbitrary units) for each network and neural event for monkey 1 starting from 5 TRs (10 s) prior to event onset until 5 TRs after event onset (mean across datasets ± SEM). (B) Evoked response values for each network and neural event for monkey 2. See Figure S3 for time courses averaged across both monkeys.

To confirm that the HRF also reflected the actual BOLD response profile in each network, we plotted the evoked BOLD response (10 s before until 10 s after event onset) averaged across sessions in both monkeys for the DMN and ventral somatomotor network by each event type (Figures 3 and S3).

#### Monkey 1

We observed a rise in the DMN BOLD signal immediately following the onset of hippocampal ripple events peaking 8 s after onset (Figure 3A). The effect of DMN BOLD signal changes after hpsigma events appeared to be anti-correlated with the DMN ripple effect, while there was no significant change in DMN BOLD signal around gamma events or ventral somatomotor network signals around any neural events in the hippocampus.

#### Monkey 2

We also observed a rise in the DMN BOLD signal immediately following the onset of hippocampal ripple events peaking at 6 s after onset, although the DMN signal was always above the baseline, i.e., above the mean BOLD signal intensity in the session (Figure 3B). DMN BOLD signal changes around hpsigma events peaked more rapidly around hpsigma event onset than the DMN changes after ripples. Additionally, there was no significant change in the DMN BOLD signal around gamma events. There was also a slight decrease in the ventral somatomotor network BOLD signal peaking around 8 s after ripple event onsets, but there were no other changes in the ventral somatomotor network BOLD signal around any hpsigma or gamma events in the hippocampus.

## Discussion

We observed a significant increase in the DMN BOLD signal following hippocampal ripples, but not following other neurophysiological events in the hippocampus. Furthermore, we did not find similar BOLD increases in a prominent ventral somatomotor network after ripples in both monkeys.

We present the first evidence, to our knowledge, of BOLD signal increases in an ongoing RSN related to hippocampal ripples. These findings are in line with a previous study [27] showing greater neocortical activations, including in regions that are part of the DMN. Crucially, these DMN changes were not observed in other prominent neocortical monkey RSNs, suggesting that correlated endogenous fluctuations in DMN regions play a privileged role in the well-known communication between the hippocampus and neocortex [3, 14, 15]. These findings are not mutually exclusive with results presented by Logothetis and colleagues [27], where voxels throughout the neocortex are activated after ripples, i.e., a fraction of voxels are transiently activated within many different neocortical regions. However, at the network level, we observe that ripples are selectively influencing endogenous RSN fluctuations that are already correlated across different neocortical regions (see Figure 1B), i.e., modulating all voxels within the DMN unitarily, but not all voxels within other RSNs.

Our results help connect seemingly disparate hypotheses about the behavioral relevance of RSNs and recent findings related to hippocampal ripples. Specifically, previous hypotheses posit that RSNs might reflect the past history of prior task activation and then potentially recapitulate this activation history in order to code information prospectively [2, 30]. These hypotheses align well with recent findings from rodent hippocampal recordings of ripples showing offline “preplay” of hippocampal place cell ensembles of locations or trajectories that had not yet been visited [31, 32]. Consequently, our data provide preliminary support that hippocampal ripples might help the DMN simulate the outcomes of prospective choices by replaying relevant memories.

The DMN we isolate with our analyses, including retrosplenial cortex, posterior cingulate, bilateral posterior medial temporal lobe (MTL), and caudal temporal parietal occipital cortex (TPOC), resembles the DMN found in previous anesthetized and awake monkey resting-state fMRI studies [28, 33]. The monkey DMN corresponds well with the MTL sub-network of the human DMN [34, 35]. However, one region missing in our DMN component when compared to humans is a large ventral medial prefrontal cortex (vmPFC) cluster, which is potentially the result of increased susceptibility artifacts.

We observed opposing or inverted responses in the DMN after hpsigma events between the two monkeys. Possible explanations for this result are that it represents normal neurophysiological variability or is a potential side effect of the anesthetic, remifentanil. However, the side-effect explanation is unlikely, since remifentanil is known to have only a negligible effect on neurovascular coupling [36, 37] and only mildly affects the time course and magnitude of neural and vascular responses [38, 39, 40]. Further evidence against the side-effect explanation comes from a recent study [41] that found no significant difference between the hippocampal theta rhythm of the anesthetized monkeys analyzed here and the unanesthetized monkey presented in [27].

Future studies that manipulate ripples with concurrent measurements at the level of whole-brain networks can move beyond our correlational results to better explore how ripples might directly modulate endogenous DMN fluctuations. One promising mechanism for how ripples could influence the DMN relates to the high amplitude of hippocampal sharp-waves (an order of magnitude larger than the amplitude of the other neural events), which co-occur with ripples, making them more likely to propagate from the hippocampus [27, 41]. Furthermore, dense hippocampal projections to the retrosplenial and posterior cingulate cortices, regions at the core of the DMN, have been found in rodents [42], macaques [43], and humans [44]. Taken together, these findings suggest that the DMN should be a primary target for propagating activity generated by hippocampal sharp-wave ripples. Neural mass models [45, 46], used with structural connectivity measurements, are ideally suited to theoretically capture the interplay between circuit-level dynamics in the hippocampus and ongoing neocortical BOLD fluctuations. These models could allow researchers to determine whether there are specific types of RSN BOLD signal changes due to hippocampal ripples versus unrelated spontaneous changes.

Our findings are a first step toward capturing the interplay between local neural events in the hippocampus and large-scale RSN dynamics. Notably, the DMN includes neocortical regions important for imagination and episodic memory, allowing for the possibility that hippocampal ripples replay past experience and help the DMN explore potential outcomes of upcoming decisions [2]. Using neural-event-triggered fMRI measurements before and after behavioral training, future studies can potentially characterize how learning modulates neural activity at both DMN and hippocampal circuit levels [3, 46].

## Experimental Procedures

### Overview

All datasets analyzed were from experiments conducted on two monkeys (E and I) originally published in [27]. All surgical and experimental procedures were approved by the local authorities (Regierungspräsidium, Tübingen Referat 35, Veterinärwesen) and were in full compliance with the guidelines of the European Community (EUVD 86/609/EEC) for the care and use of laboratory animals. Experiments were carried out in two male rhesus monkeys (*Macaca mulatta*). Neural responses in hippocampal CA1 during resting-state experiments were collected in 50 fMRI experiments/sessions (25 for each monkey) that lasted 10 min each. For details on surgical procedures, see [27]. See Supplemental Experimental Procedures for details on detection of hippocampal ripples and other neural events, maintenance of anesthesia, MRI acquisition, and fMRI preprocessing/statistical analyses.

### Resting-State Network Analysis

Spatial ICA, a technique that extracts maximally independent patterns of coherent fMRI activity [47], was applied to each single dataset by means of the GIFT toolbox (http://icatb.sourceforge.net). The estimation of the number of independent components (ICs) was performed using the minimum description length criterion [47]. After reduction of dimensionality by means of principal-component analysis (accounting for at least 99.9% explained variance), ICs were retrieved by means of the FastICA algorithm, with a deflation approach and hyperbolic tangent (*tanh*) nonlinearity [48]. Each fMRI IC consisted of a waveform and a spatial map: the waveform corresponded to the time course of the specific pattern, whereas the associated spatial map expressed the intensity of activity across voxels. To display voxels contributing most strongly to a particular IC and allow intersubject comparison, we scaled the intensity values in each map to Z scores. To extend the ICA analysis from single to multiple datasets, we used the self-organizing group ICA (sogICA) method [48] to sort the ICs extracted from different fMRI datasets and subsequently average them to generate a single IC dataset. SogICA was applied according to a two-stage procedure: first to IC datasets from the same subject for the creation of a representative single-subject IC dataset (within-subject analysis), and then to single-subject IC datasets for the creation of a group-level IC dataset (across-subject analysis). For each sogICA procedure, the IC clusters with relative consistencies ≤ 50% or that were spatially correlated at r > 0.20 with white matter or CSF patterns (as available in SPM5.0) were excluded from further analyses. The IC clusters obtained at the second level of sogICA were classified as resting-state networks (RSNs).

We performed hierarchical cluster analysis on the entire set of monkey RSNs in common space [49]. To characterize the clustering, we used the spatial correlation as a similarity metric and used the average linkage function. After the creation of the dendrogram, we selected the cutoff value for the graph yielding the maximum number of clusters in both monkeys. This resulted in the definition of single- or two-element clusters. To characterize the reliability of each cluster, we grouped the spatial correlations between the RSNs inside the cluster (intracluster correlations) and those between the RSNs of the cluster and all other RSNs (extracluster correlations). We statistically compared intracluster and extracluster correlations by means of the Mann-Whitney test, thus obtaining a quantitative measure of the cluster reliability. We present RSNs in each subject by averaging the signals across the voxels of the network map (threshold at Z > 2). In monkey 1, both the ventral somatomotor and DMN components were present in all 25 datasets. In monkey 2, the ventral somatomotor network component was present in all 25 datasets, while the DMN component was present in 22 datasets. In monkey 2, one dataset did not have any occurrence of gamma events; otherwise there were occurrences of each neural event in every session. Consequently, there were 25 ventral somatomotor and DMN datasets analyzed with monkey 1 and 24 ventral somatomotor and 21 DMN datasets analyzed with monkey 2.

## Author Contributions

N.K.L. conceived and designed the experiments. R.K. and G.D. conceived the hypothesis and corresponding analyses. N.K.L. and Y.M. performed the experiments. D.M, M.H.A., and R.H. provided analysis tools. R.K. analyzed the data and wrote the manuscript with input from all of the authors.

## Figures and Tables

**Table 1:** t Values of RSN BOLD Signal Changes after Each Neural Event

	Hpsigma	Gamma	Ripple
**Monkey 1**			

Default	−3.55^∗^	0.614	5.66^∗∗^
Somatomotor	0.561	0.049	1.22

**Monkey 2**			

Default	3.25^∗^	0.172	9.41^∗∗^
Somatomotor	−1.86	1.43	−3.06^∗^

^∗^p ≤ 0.01; ^∗∗^p ≤ 0.001.

## Supplemental Information

Document S1. Figures S1–S3, Table S1, and Supplemental Experimental Procedures

Document S2. Article plus Supplemental Information
