# Supplementary material for: Hippocampal Sharp-Wave Ripples Influence Selective Activation of the Default Mode Network
Source: Curr Biol. 2016 Mar 7;26(5):686–91. doi: 10.1016/j.cub.2016.01.017 (PMC4791429; doi:10.1016/j.cub.2016.01.017)
Supplement: Document S1. Figures S1–S3, Table S1, and Supplemental Experimental Procedures [file mmc1.pdf]

Current Biology, Volume *26*

Supplemental Information

Hippocampal Sharp-Wave Ripples Influence Selective Activation of the Default Mode Network

Raphael Kaplan, Mohit H. Adhikari, Rikkert Hindriks, Dante Mantini, Yusuke Murayama, Nikos K. Logothetis, and Gustavo Deco

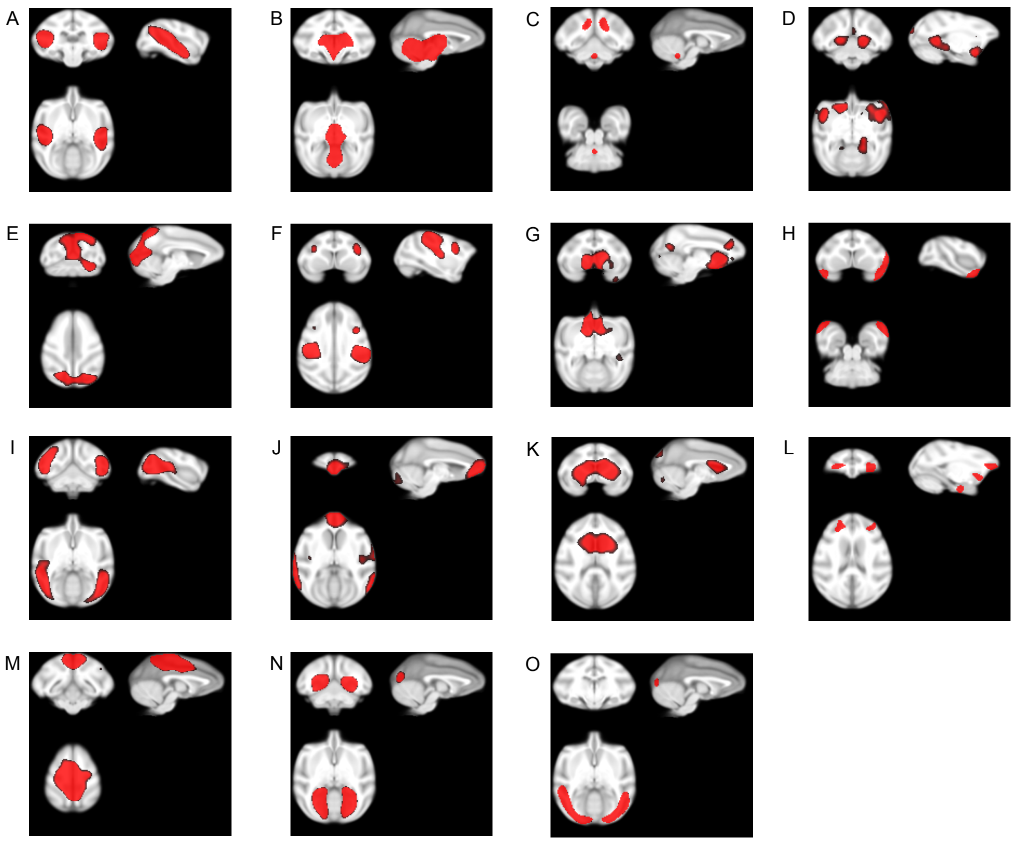

Figure S1, related to Figure 1: Images of all remaining independent components (ICs) identified as resting-state networks present in both monkeys using ICA. A. Occipitotemporal B. Subcortical network including thalamus C. Parieto-cerebellar D. Hippocampal-Prefrontal E. Visual F. Frontoparietal G. Anterior Cingulate H. Temporal pole I. Parieto-occipital J. Frontal pole-temporoparietal K. Basal Ganglia L. Amygdala-Orbitofrontal M. Posterior Cingulate N. Primary Visual O. Middle Visual. Networks shown at slices most representative of the correlation pattern that network identification was based. All images thresholded at Z-score>2 and overlaid on a composite structural from the UWRMAC-DTI271 atlas space.

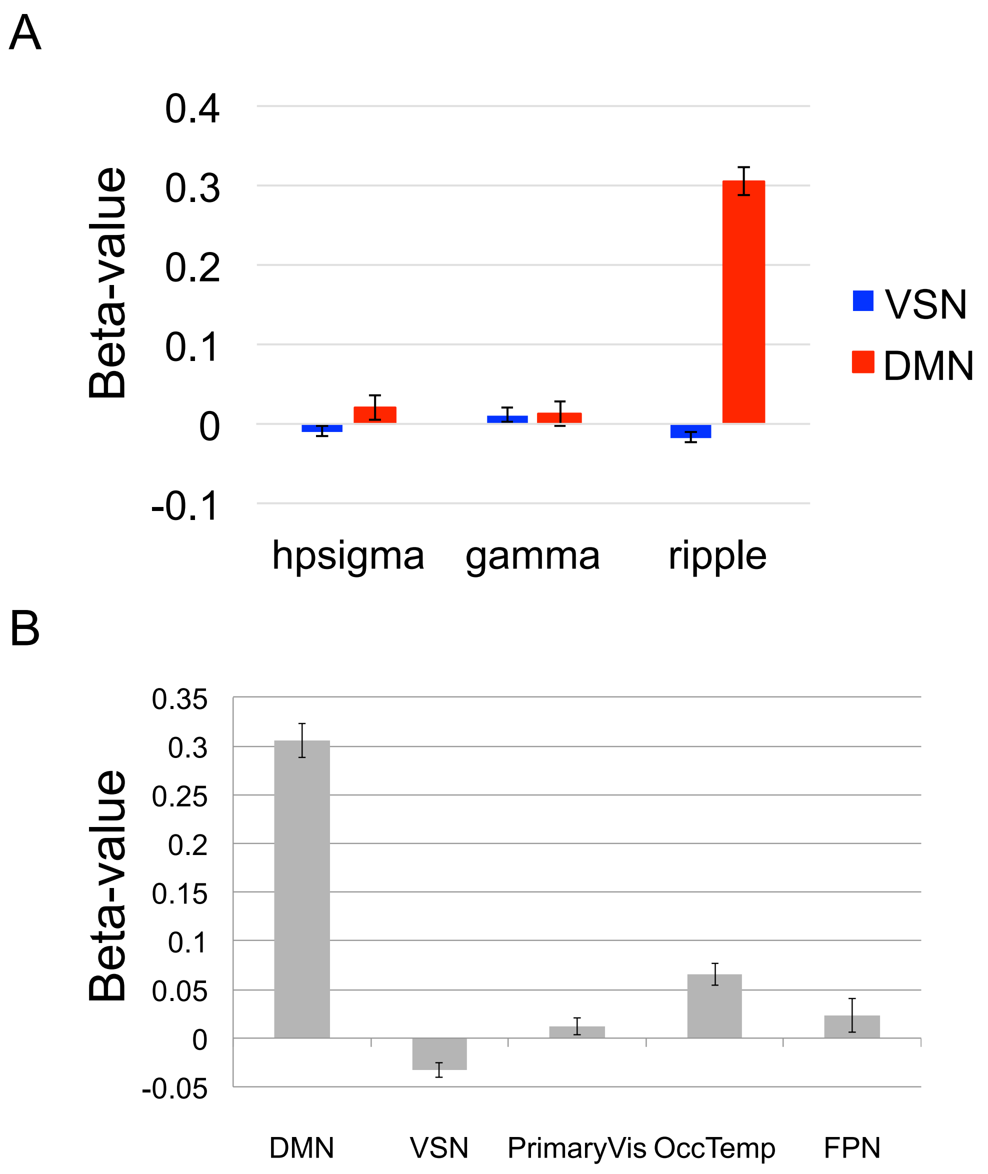

Figure S2, related to Figure 2 and Figure S1: Group Average of fMRI signal changes after ripples in DMN versus other RSNs. A. Beta-values for VSN and DMN after each neural event averaged across both monkeys (mean ± SEM). B. Beta-values after the onset of ripples for DMN, VSN and three other neocortical resting-state networks (Primary Visual, Occipitotemporal, and Frontoparietal) averaged across both monkeys (mean ± SEM). The three other RSNs were chosen, since they were the only neocortical RSNs with ICs present in over half of the 25 datasets in both monkeys. Primary Visual Network ICs (see Figure S1N for anatomical image) were observable in 16 datasets in Monkey 1 and 20 datasets in Monkey 2, Occipitotemporal Network (see Figure S1A for anatomical image) ICs were observable in 24 datasets in Monkey 1 and 14 datasets in Monkey 2, and Frontoparietal Network ICs (see Figure S1F for anatomical image) were observable in 19 datasets in Monkey 1 and 13 datasets in Monkey 2.

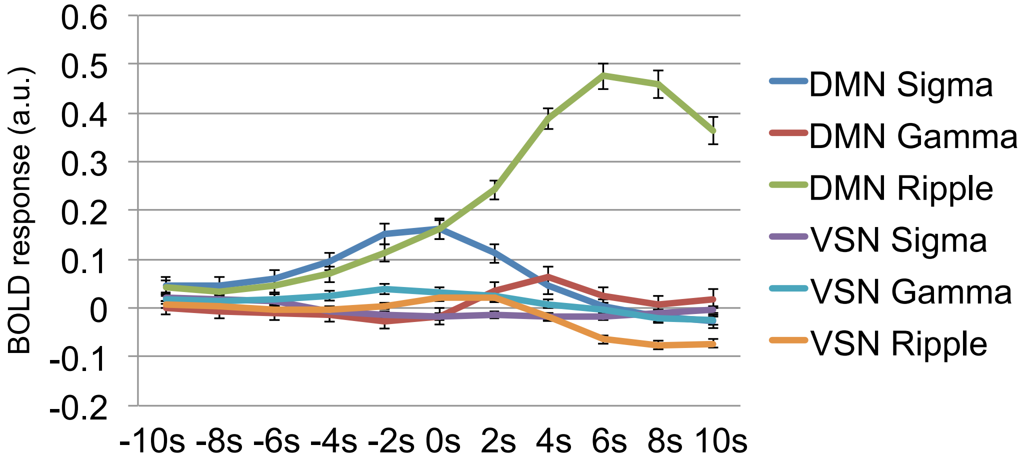

Figure S3, related to Figure 3: Time course of hippocampal neural events in DMN and Ventral Somatomotor Network group average. Evoked responses (signal amplitude presented in arbitrary units) for each network and neural event averaged across both monkeys starting from 5 TRs prior to event onset until 5 TRs after event onset (mean across datasets ± SEM).

Monkey 1	Gamma	Ripple
PCC-Left Hippocampus	.847**	.662**
PCC-Right Hippocampus	.912**	.728**
Monkey 2	Gamma	Ripple
PCC-Left Hippocampus	.267	.528*
PCC-Right Hippocampus	.249	.37

****p<.001;*p<.05**

Table S1, related to Table 1: Correlation between default mode network and hippocampal beta-values.

Pearson correlation coefficient values between individual voxels in left and right hippocampus with a voxel in a key DMN hub, the posterior cingulate cortex (PCC) after gamma and ripple events. Gamma events were used as a control event, since they were not found to significantly influence DMN activity. We found in Monkey 1 that both hippocampal voxels were correlated with the PCC after ripples and gamma events. However in Monkey 2, we only observed a significant correlation between the left hippocampus and DMN after ripples. Beta-values were calculated using the same HRF presented in all other analyses.

Supplemental Experimental Procedures

Ripple and Neural Event Detection

The detection of ripples was made using previously described methodology [S1-3], but with refinements in order to permit classification of events detected by significant power changes across a broad frequency spectrum. Electrode tips were first visually classified into pyramidal cell layer (pl) and stratum radiatum (sr) channels on the basis of their activity patterns (e.g. complex spike characteristics, fast oscillations, SPW-like signal amplitude-deflections. Clear sharp-waves and ripple oscillations could be visibly detected by inspecting the denoised comprehensive or LFP (0.05-250 Hz) signals of each electrode, together with their (80-180 Hz) band-pass-filtered derivative. To detect events, the LFP signal from the pl channels was first filtered between 5 and 200 Hz, rectified, low-pass filtered at 20 Hz with a 4th order Butterworth filter, and finally z-score normalized. Epochs during which the normalized signal exceeded a 3.5 standard deviation threshold were initially considered candidate oscillatory events. After off-line examination of the spectra, the (5-200 Hz) window was reduced to (10-180 Hz), capturing all significant changes occurring during the events in these experiments [S4]. Following the detection of candidate events, spectral analysis using multitaper FFT [S5] was performed on the LFP signal, and the spectra were separated into clusters using the non-negative matrix factorization (NMF) process [S6], an unsupervised feature extraction algorithm permitting the decomposition of multivariate data into a user-defined number of clusters. NMF was used with 10 factorization repetitions. For each spectral cluster, we examined how well a single factor of the NMF results could explain the power spectrum. Factorization quality was quantified using the signal-to-noise (SNR) of the factor, defined as the ration of the sum of the squared power spectrum values to the sum of the squared difference of the spectrum, from its projection on that single factor. Each event was then associated to the factor with the highest SNR, and only "pure" events (i.e. events with almost unimodal spectral distribution) associated to a factor with SNR>3 were selected for further analysis and event-triggered BOLD fMRI. This procedure yielded three robust and distinct clusters in the frequency ranges of 8-22 Hz, 25-75 Hz, and 80-180 Hz, labelled hpsigma, gamma, and ripple ranges respectively. Unlike [S4], which implemented time-course fMRI analysis, where adequate temporal spacing was important, we performed event-related regression analysis, where temporal spacing >1s was not important. Consequently, we did not eliminate neural events occurring within less than one second of each other.

Maintenance of Anesthesia during physiology and fMRI experiments

Experimental recordings were conducted while the animals were under general anesthesia. During the fMRI experiments anesthesia was maintained with remifentanil (0.5-2 µg/kg/min) in combination with a fast-acting paralytic, mivacurium chloride (5-7mg/kg/hr). Anesthesia dosages were determined by measuring stress hormones and were selected to ensure unaffected physiological responses at normal catecholamine concentrations [S4, 7]. Notably, remifentanil is an ultra fast acting μ opioid receptor agonist that has no significant effect on neurovascular activity [S8-9]. Numerous studies [S10-12], including ones using the datasets analyzed in this paper [S4, 13], have shown that remifentanil negligibly affects the magnitude and time course of neural and vascular responses, including the hippocampal LFP [S13]. Additionally, the physiological state of the animal was monitored continuously and maintained tightly within normal limits. Body temperature was maintained at 38-39°C, and end-tidal CO_2 and oxygen saturation were kept constant at 33 mm Hg and over 95%, respectively [S4].

MRI Acquisition

Experiments were conducted in a vertical 4.7 Tesla scanner (Bruker BioSpin, Ettlingen, Germany) with a 40 cm diameter bore. The system has a 50mT/m (180 µs rise time) actively shielded gradient coil (Bruker, BGA26) with an inner diameter of 26 cm. We used a custom-made chair to position the monkey in the magnet and a customized quadrature volume radiofrequency (RF) coil. At the beginning of the experiment, we acquired anatomical images to precisely confirm the electrode position. Coronal sections parallel to the electrode were acquired using a TURBO-RARE sequence with the following parameters: field of view 96 mm x 96 mm, matrix 384x256, in-plane resolution 0.25 mm x 0.375 mm, rare factor 8, effective TE of 60 ms, TR of 3000 ms, BW 38 kHz, 15 slices, slice thickness 0.5 mm and 4 averages. 22 axial slices covering most of the brain were acquired. BOLD activity from these slices was acquired at a TR of 2 seconds with two-shot GE-EPI images (TR/TE=1000/20ms, bandwidth=150 kHz, FA=53o, FOV=96x96mm, matrix=96x96, 2mm slice thickness). In a given experiment, 25 fMRI files (sessions) were acquired containing 300 volumes of 2

seconds duration with spontaneous activity within 4.5 hours. T2-weighted RARE images with the same FOV were obtained using a matrix of 256x256, rare factor 8, effective TE of 60 ms, TR of 5000 ms, BW 42 kHz, and 4 averages. MRI data were analyzed off-line using our own software developed in MATLAB.

fMRI preprocessing

fMRI data preprocessing was performed with the SPM8 software package (www.fil.ion.ucl.ac.uk/spm) running under MATLAB (MathWorks). The first four volumes were discarded to allow for T1 equilibration. Images were coregistered to the anatomical image and spatial normalized to UWRMAC-DTI271 atlas space [S14]. Lastly, data was spatially smoothed with a Gaussian kernel at 5 mm full-width-half-maximum.

Statistical Analyses

Using custom Matlab scripts, we convolved 1117 hpsigma, 823 gamma, and 1720 ripple events in Monkey 1 and 887 hpsigma, 917 gamma, and 911 ripple events in Monkey 2 with the normalized canonical hemodynamic response function (HRF) from SPM8. We subsequently used events as regressors in a standard event-related design. This design gave us a 2x3 within-session repeated measures ANOVA of network (ventral somatomotor and default mode) by event (hpsigma, gamma, and ripple) for each monkey. We then report the corresponding one-sample and paired t-test comparisons after testing for the interaction.

For the time course analysis, we plotted the evoked BOLD response at each TR ranging from 5 TRs before until 5 TRs after the onset of each neural event without fitting to any HRF.

Supplemental References

S1. Buzsáki, G., Horvath, Z., Urioste, R., Hetke, J., Wise, K. (1992). High-frequency network oscillation in the hippocampus. Science *256*, 1025-1027.

S2. Skaggs, W.E., McNaughton, B.L., Permenter, M., Archibeque, M., Vogt, J., Amaral, D.G., and Barnes, C.A. (2007). EEG sharp waves and sparse ensemble unit activity in the macaque hippocampus. J. Neurophysiol. *98*, 898-910.

S3. Sullivan, D., Csicvari, J., Mizuseki, K., Montgomery, S., Diba, K., Buzsaki G. (2011). Relationships between hippocampal sharp waves, ripples, and fast gamma oscillation: influence of dentate and entorhinal cortical activity. J. Neurosci. *31*, 8605-8616.

S4. Logothetis, N.K., Eschenko, O., Murayama, Y., Augath, M., Steudel, T., Evrard, H.C., Besserve, M., and Oeltermann, A. (2012). Hippocampal-cortical interaction during periods of subcortical silence. Nature *491*, 547-553.

S5. Mitra, P.P. and Pesaran, B. (1999). Analysis of dynamic brain imaging data. Biophys. J. *76*, 691-708.

S6. Lee, D.D. and Seung, H.S. (1999) Learning the parts of objects by non-negative matrix factorization. Nature *401*, 788-791.

S7. Canals, S.G., Beyerein, M., Merkle, H., Logothetis, N.K. (2009). Functional MRI Evidence for LTP-Induced Neural Network Reorganization. Curr. Bio. *19*, 398-403.

S8. Wise, R.G., Rogers, R., Painter, D., Bantick, S., Ploghaus, A., Williams, P., Rapeport, G., Tracey, I. (2002). Combining fMRI with a pharmacokinetic model to determine which brain areas activated by painful stimulation are specifically modulated by remifentanil. Neuroimage *16*, 999-1014.

S9. Pattinson, K.T., Rogers, R. Mayhew, S.D., Tracey, I., Wise, R.G. (2007). Pharmacological FMRI: measuring opioid effects on the BOLD response to hypercapnia. J. Cereb. Blood Flow. Metab. *27*, 414-423.

S10. Goense, J.B. and Logothetis, N.K. (2008). Neurophysiology of the BOLD fMRI signal in awake monkeys. Curr. Bio. *18*, 631-640.

S11. Logothetis, N.K., Guggenberger, H., Peled, S., Pauls, J. (1999). Functional imaging of the monkey brain. Nat. Neurosci. *2*, 555-562.

S12. Logothetis, N.K., Pauls, J., Augath, M., Trinath, T., Oeltermann, A. (2001) Neurophysiological investigation of the basis of the fMRI signal. Nature *412*, 150-7.

S13. Ramirez-Villegas, J.F., Logothetis, N.K., Besserve, M. (2015). Diversity of sharp-wave-ripple LFP signatures reveals differentiated brain-wide dynamical events. Proc. Natl. Acad. Sci. U.S.A. *112*, E6379-6387.

S14. Adluru, N., Zhang, H., Fox, A.S., Shelton, S.E., Ennis, C.M., Bartosic, A.M., Oler, J.A., Tromp, D.P.M., Zakszewski, E., Gee, J.C., Kalin, N.H., and Alexander, A.L. (2012). A Diffusion Tensor Brain Template for Rhesus Macaques. Neuroimage *59*, 306-318
