# Supplementary material for: Hippocampal Sharp-Wave Ripples Influence Selective Activation of the Default Mode Network
Source: Curr Biol. 2016 Mar 7;26(5):686–91. doi: 10.1016/j.cub.2016.01.017 (PMC4791429; doi:10.1016/j.cub.2016.01.017)
Supplement: Document S2. Article plus Supplemental Information [file mmc2.pdf]

Report

Current Biology

Hippocampal Sharp-Wave Ripples Influence Selective Activation of the Default Mode Network

Highlights

- Behavioral relevance of offline fluctuations in the default mode network is unclear
- Hippocampal sharp-wave ripples during rest are linked with memory consolidation
- Default mode network signal increases after ripples, but not other hippocampal events
- Other neocortical resting-state networks do not show similar changes after ripples

Authors

Raphael Kaplan, Mohit H. Adhikari, Rikkert Hindriks, Dante Mantini, Yusuke Murayama, Nikos K. Logothetis, Gustavo Deco

Correspondence

raphael.kaplan.09@ucl.ac.uk

In Brief

Kaplan et al. show that offline fMRI signal fluctuations throughout the default mode network (DMN), which includes neocortical regions important for episodic memory, are uniformly influenced by hippocampal sharp-wave ripples. These results relate ongoing DMN fluctuations to the consolidation of past experience and preparation for future behavior.

Kaplan et al., 2016, Current Biology *26*, 686–691
March 7, 2016 ©2016 The Authors
http://dx.doi.org/10.1016/j.cub.2016.01.017

Current Biology
Report

Hippocampal Sharp-Wave Ripples Influence Selective Activation of the Default Mode Network

Raphael Kaplan,[1,2,*] Mohit H. Adhikari,[2] Rikkert Hindriks,[2] Dante Mantini,[3,4] Yusuke Murayama,[5] Nikos K. Logothetis,[5,6] and Gustavo Deco[2,7]
[1]Wellcome Trust Centre for Neuroimaging, UCL Institute of Neurology, University College London, 12 Queen Square, London WC1N 3BG, UK
[2]Center for Brain and Cognition, Departament de Tecnologies de la Informació I les Comunicacions, Universitat Pompeu Fabra, Roc Boronat 138, 08018 Barcelona, Spain
[3]Department of Health Sciences and Technology, ETH Zurich, 8057 Zurich, Switzerland
[4]Movement Control and Neuroplasticity Research Group, KU Leuven, 3001 Leuven, Belgium
[5]Max Planck Institute for Biological Cybernetics, 72076 Tübingen, Germany
[6]Imaging Science and Biomedical Engineering, University of Manchester, Manchester M13 9PT, UK
[7]Institució Catalana de la Recerca i Estudis Avançats (ICREA), Universitat Pompeu Fabra, Passeig Lluís Companys 23, Barcelona 08010, Spain
*Correspondence: raphael.kaplan.09@ucl.ac.uk
http://dx.doi.org/10.1016/j.cub.2016.01.017
This is an open access article under the CC BY license (http://creativecommons.org/licenses/by/4.0/).

SUMMARY

The default mode network (DMN) is a commonly observed resting-state network (RSN) that includes medial temporal, parietal, and prefrontal regions involved in episodic memory [1–3]. The behavioral relevance of endogenous DMN activity remains elusive, despite an emerging literature correlating resting fMRI fluctuations with memory performance [4, 5]—particularly in DMN regions [6–8]. Mechanistic support for the DMN's role in memory consolidation might come from investigation of large deflections (sharp-waves) in the hippocampal local field potential that co-occur with high-frequency (>80 Hz) oscillations called ripples—both during sleep [9, 10] and awake deliberative periods [11–13]. Ripples are ideally suited for memory consolidation [14, 15], since the reactivation of hippocampal place cell ensembles occurs during ripples [16–19]. Moreover, the number of ripples after learning predicts subsequent memory performance in rodents [20–22] and humans [23], whereas electrical stimulation of the hippocampus after learning interferes with memory consolidation [24–26]. A recent study in macaques showed diffuse fMRI neocortical activation and subcortical deactivation specifically after ripples [27]. Yet it is unclear whether ripples and other hippocampal neural events influence endogenous fluctuations in specific RSNs—like the DMN—unitarily. Here, we examine fMRI datasets from anesthetized monkeys with simultaneous hippocampal electrophysiology recordings, where we observe a dramatic increase in the DMN fMRI signal following ripples, but not following other hippocampal electrophysiological events. Crucially, we find increases in ongoing DMN activity after ripples, but not in other RSNs. Our results relate endogenous DMN fluctuations to hippocampal ripples, thereby linking network-level resting fMRI fluctuations with behaviorally relevant circuit-level neural dynamics.

RESULTS

We present novel analyses conducted on fMRI datasets from two anesthetized macaques used in a prior study by Logothetis and colleagues [27], where we ascertained whether there were changes at the level of whole-brain resting-state networks (RSNs) after hippocampal hpsigma (8–22 Hz), gamma (25–75 Hz), or ripple (80–180 Hz) events. We implemented a recently developed technique that uses spatial independent component analysis (ICA) to define correlated fMRI signal fluctuations measured across multiple scan experiments/sessions and subjects into component brain networks [28, 29]. Analyzing 25 fMRI sessions each lasting 10 min in both subjects, we isolated the macaque equivalent of the default mode network (DMN) and compared it to the most robustly observed RSN across sessions and monkeys in our data, the ventral somatomotor network. First, we investigated whether there were positive DMN blood-oxygen-level-dependent (BOLD) signal responses after hippocampal ripples and whether these responses also occurred after the onset of hippocampal hpsigma and gamma events. Second, we investigated whether these three different hippocampal events also co-occurred with BOLD signal fluctuations in the ventral somatomotor network, a RSN not implicated in hippocampal-dependent memory consolidation. Consequently, we could determine whether RSN responses were network- and neural-event specific.

We first used spatial ICA on fMRI experiments/sessions from two monkeys to define RSNs of brain areas showing correlated fMRI activity. After performing a cluster analysis to establish the topological correspondence of RSNs across sessions and monkeys, we isolated the DMN and ventral somatomotor network (Figure 1; see Figure S1 for remaining RSNs). fMRI independent component (IC) time courses for the DMN and ventral

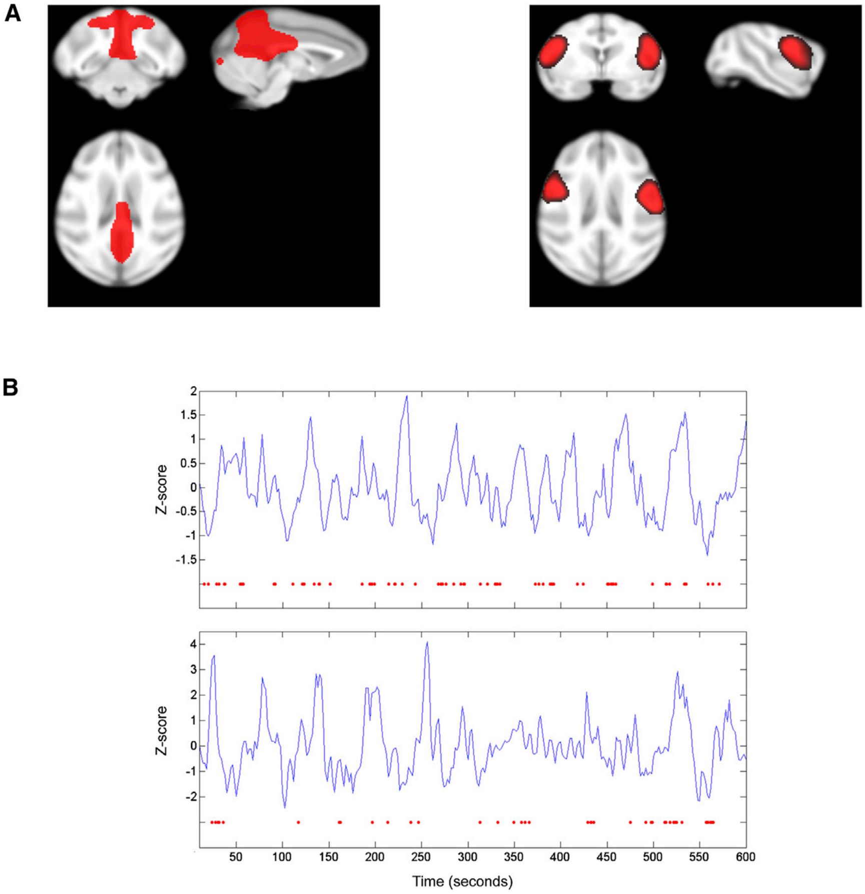

Figure 1. Default Mode and Ventral Somatomotor Resting-State Networks

(A) Group-level fixed-effects image of default mode network (DMN; left) and ventral somatomotor network (VSN; right) in two rhesus monkeys. Networks are shown at slices most representative of the correlation pattern on which network identification was based. Images were statistically thresholded at Z > 2 and overlaid on a composite structural from the UWRMAC-DTI271 atlas space. See Figure S1 for other resting-state independent components (ICs) present in both monkeys.

(B) DMN time course for a representative 10 min session in monkey 1 (top plot) and monkey 2 (bottom plot), where red dots below represent the onset of hippocampal ripple events. Representative sessions were chosen based on closeness to mean IC rank out of all present ICs for a given session (monkey 1: mean = 4.04, displayed = 3; monkey 2: mean = 6.64, displayed = 7) and ripple amount (monkey 1: mean = 68.8, displayed = 69; monkey 2: mean = 41.2, displayed = 39).

somatomotor network were then aligned to the onset of hpsigma (monkey 1: n_{events} = 1117; monkey 2: n_{events} = 887), gamma (monkey 1: n_{events} = 823; monkey 2: n_{events} = 917), and ripple (monkey 1: n_{events} = 1720; monkey 2: n_{events} = 911) events, and their averages were convolved with a hemodynamic response function (HRF). These average event-related BOLD signals were subsequently used as regressors in a standard event-related fMRI design. We report statistics for each monkey across sessions. To determine whether the HRF accurately captured the BOLD response to each event, we also plotted the evoked BOLD response for both RSNs without any fitting.

Effect of Neural Events on RSNs

Monkey 1

Using a 2 × 3 within-session repeated-measures ANOVA for network by neural event, we found a significant interaction ($F(2,23) = 11.9$, $p < 0.001$; Figure 2A). We also found a main effect for neural event ($F(2,23) = 14.9$, $p < 0.001$) but not network ($F(1,24) = 0.210$, $p = 0.651$). This interaction was driven by positive DMN BOLD responses after ripples ($t(24) = 5.26$, $p < 0.001$). Paired t tests revealed that there were significantly higher DMN activations after ripples compared to hpsigma ($t(24) = 5.99$, $p < 0.001$) or gamma ($t(24) = 2.50$, $p = 0.020$) events. Additionally, there was significantly higher DMN versus ventral somatomotor network activity ($t(24) = 3.62$, $p = 0.001$) after ripples.

For the other neural events, we found a significant decrease in DMN activity ($t(24) = -3.55$, $p = 0.002$) after hpsigma events. DMN activity after gamma events was significantly lower for hpsigma versus gamma events ($t(24) = -2.45$, $p = 0.022$). DMN activity was also significantly lower ($t(24) = -3.05$, $p = 0.006$) compared to ventral somatomotor activity after hpsigma events. Otherwise, we observed no significant differences between events for ventral somatomotor network activity. Furthermore, there were no other significant changes versus baseline in either network for the other neural events. See Table 1 for a complete listing of one-sample t values for DMN and ventral somatomotor network activity after hpsigma, gamma, and ripple events and Table S1 for DMN correlations with left and right hippocampus after ripples.

Monkey 2

Using a 2 × 3 within-session repeated-measures ANOVA for network by neural event, we found a significant interaction ($F(2,18) = 36.2$, $p < 0.001$; Figure 2B). We also found a main effect for both neural event ($F(2,18) = 14.3$, $p < 0.001$) and network ($F(1,19) = 59.1$, $p < 0.001$). Once again, this effect was related to DMN increases after ripple events ($t(21) = 9.41$, $p < 0.001$). In follow-up paired t tests, there was significantly higher DMN activity after ripples compared to hpsigma ($t(21) = 6.48$, $p < 0.001$) or gamma ($t(20) = 7.42$, $p < 0.001$) events. Additionally, there was significantly higher DMN versus ventral somatomotor network activity after ripples ($t(21) = 11.6$, $p < 0.001$).

Converse to monkey 1, there was a significant increase in DMN activity after hpsigma events ($t(21) = 3.25$, $p = 0.004$). DMN activity after hpsigma events was significantly higher ($t(20) = 2.26$, $p = 0.035$) than after gamma events and higher ($t(21) = 3.86$, $p = 0.001$) than ventral somatomotor activity after hpsigma events. Additionally, there was a significant decrease in ventral somatomotor activity after the onset of ripple events

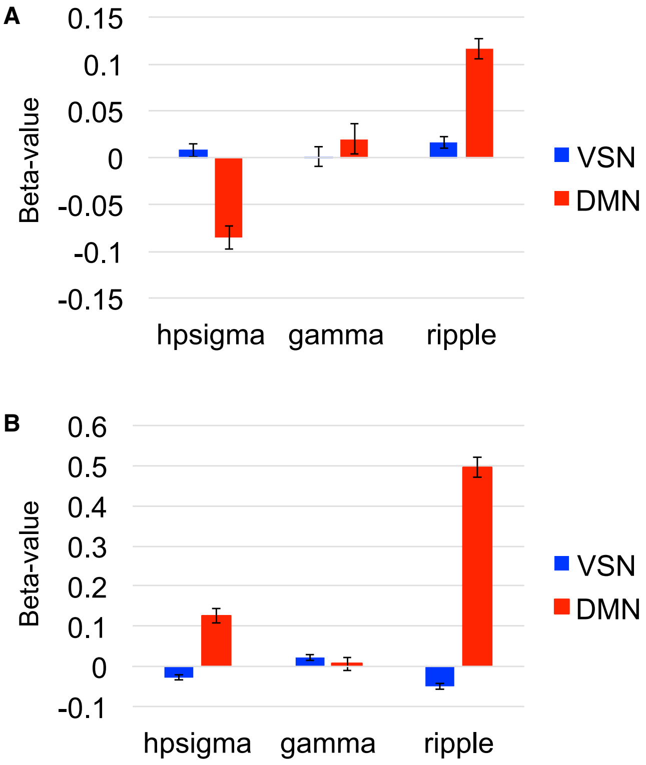

Figure 2. Influence of Hippocampal Neural Events on DMN and VSN

(A) Beta values for each network and neural event for monkey 1 (mean ± SEM). There were significantly higher DMN activations after ripples compared to hpsigma ($t(24) = 5.99$, $p < 0.001$) or gamma ($t(24) = 2.50$, $p = 0.020$) events. Additionally, there was significantly higher DMN versus VSN activity ($t(24) = 3.62$, $p = 0.001$) after ripples. There was also a significant decrease in DMN activity ($t(24) = -3.55$, $p = 0.002$) after hpsigma events, also when compared to gamma events ($t(24) = -2.45$, $p = 0.022$). DMN activity was also significantly lower ($t(24) = -3.05$, $p = 0.006$) compared to VSN activity after hpsigma events.

(B) Beta values for each network and neural event for monkey 2 (mean ± SEM). There was significantly higher DMN activity after ripples compared to hpsigma ($t(21) = 6.48$, $p < 0.001$) or gamma ($t(20) = 7.42$, $p < 0.001$) events. Additionally, there was significantly higher DMN versus VSN activity after ripples ($t(21) = 11.6$, $p < 0.001$). Converse to monkey 1, there was a significant increase in DMN activity after hpsigma events ($t(21) = 3.25$, $p = 0.004$), which was significantly higher ($t(20) = 2.26$, $p = 0.035$) than after DMN gamma events and also higher ($t(21) = 3.86$, $p = 0.001$) than VSN activity after hpsigma events. There was a significant decrease in ventral somatomotor activity after the onset of ripple events ($t(24) = -3.06$, $p = 0.006$).

See Figure S2 for mean plots averaged across both monkeys, along with plots showing effect of ripples on other neocortical RSNs.

($t(24) = -3.06$, $p = 0.006$), but not after any other events (see Table 1 for a complete listing of t values for both the response of both networks to each event and Table S1 for DMN correlations with left and right hippocampus after ripples). Lastly, there was significantly lower ventral somatomotor network activity after ripples than after gamma events ($t(23) = 3.04$, $p = 0.006$), while there was a trend ($t(23) = 2.07$, $p = 0.050$) for higher ventral somatomotor activity for gamma versus hpsigma events. See Figure S2A of the aforementioned effects averaged across both monkeys. To add further specificity to our findings, we investigated three other neocortical RSNs with ICs that were less robust than the DMN and VSN but still observable in more than half of the datasets in both monkeys: the primary visual, occipitotemporal, and frontoparietal networks (Figure S2B). Notably, we observed only negligible BOLD changes after ripples in these three RSNs across both monkeys (Figure S2B).

Table 1. t Values of RSN BOLD Signal Changes after Each Neural Event

	Hpsigma	Gamma	Ripple
Monkey 1			
Default	−3.55*	0.614	5.66**
Somatomotor	0.561	0.049	1.22
Monkey 2			
Default	3.25*	0.172	9.41**
Somatomotor	−1.86	1.43	−3.06*

*$p \leq 0.01$; **$p \leq 0.001$.

Temporal Profile of Effect of Neural Events on RSNs

To confirm that the HRF also reflected the actual BOLD response profile in each network, we plotted the evoked BOLD response (10 s before until 10 s after event onset) averaged across sessions in both monkeys for the DMN and ventral somatomotor network by each event type (Figures 3 and S3).

Monkey 1

We observed a rise in the DMN BOLD signal immediately following the onset of hippocampal ripple events peaking 8 s after onset (Figure 3A). The effect of DMN BOLD signal changes after hpsigma events appeared to be anti-correlated with the DMN ripple effect, while there was no significant change in DMN BOLD signal around gamma events or ventral somatomotor network signals around any neural events in the hippocampus.

Monkey 2

We also observed a rise in the DMN BOLD signal immediately following the onset of hippocampal ripple events peaking at 6 s after onset, although the DMN signal was always above the baseline, i.e., above the mean BOLD signal intensity in the session (Figure 3B). DMN BOLD signal changes around hpsigma events peaked more rapidly around hpsigma event onset than the DMN changes after ripples. Additionally, there was no significant change in the DMN BOLD signal around gamma events. There was also a slight decrease in the ventral somatomotor network BOLD signal peaking around 8 s after ripple event onsets, but there were no other changes in the ventral somatomotor network BOLD signal around any hpsigma or gamma events in the hippocampus.

DISCUSSION

We observed a significant increase in the DMN BOLD signal following hippocampal ripples, but not following other neurophysiological events in the hippocampus. Furthermore, we did not find similar BOLD increases in a prominent ventral somatomotor network after ripples in both monkeys.

We present the first evidence, to our knowledge, of BOLD signal increases in an ongoing RSN related to hippocampal ripples. These findings are in line with a previous study [27] showing greater neocortical activations, including in regions that are part of the DMN. Crucially, these DMN changes were not observed in

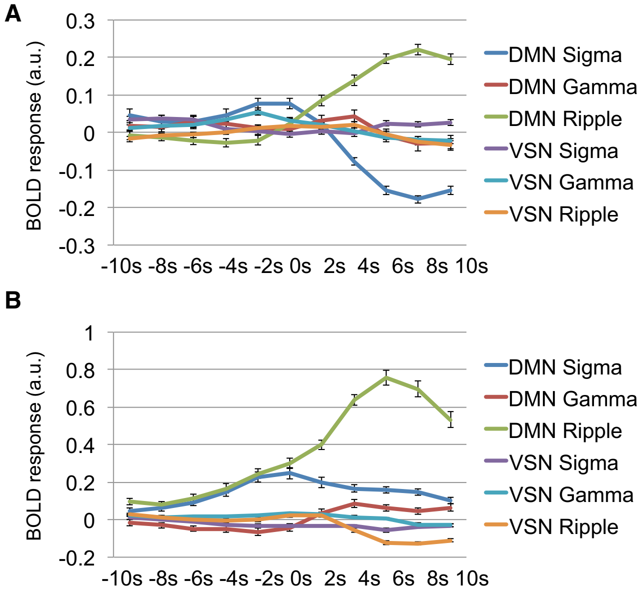

Figure 3. Time Course of Hippocampal Neural Events in DMN and VSN

(A) Evoked response values (signal amplitude presented in arbitrary units) for each network and neural event for monkey 1 starting from 5 TRs (10 s) prior to event onset until 5 TRs after event onset (mean across datasets ± SEM).

(B) Evoked response values for each network and neural event for monkey 2. See Figure S3 for time courses averaged across both monkeys.

other prominent neocortical monkey RSNs, suggesting that correlated endogenous fluctuations in DMN regions play a privileged role in the well-known communication between the hippocampus and neocortex [3, 14, 15]. These findings are not mutually exclusive with results presented by Logothetis and colleagues [27], where voxels throughout the neocortex are activated after ripples, i.e., a fraction of voxels are transiently activated within many different neocortical regions. However, at the network level, we observe that ripples are selectively influencing endogenous RSN fluctuations that are already correlated across different neocortical regions (see Figure 1B), i.e., modulating all voxels within the DMN unitarily, but not all voxels within other RSNs.

Our results help connect seemingly disparate hypotheses about the behavioral relevance of RSNs and recent findings related to hippocampal ripples. Specifically, previous hypotheses posit that RSNs might reflect the past history of prior task activation and then potentially recapitulate this activation history in order to code information prospectively [2, 30]. These hypotheses align well with recent findings from rodent hippocampal recordings of ripples showing offline “preplay” of hippocampal place cell ensembles of locations or trajectories that had not yet been visited [31, 32]. Consequently, our data provide preliminary support that hippocampal ripples might help the DMN simulate the outcomes of prospective choices by replaying relevant memories.

The DMN we isolate with our analyses, including retrosplenial cortex, posterior cingulate, bilateral posterior medial temporal lobe (MTL), and caudal temporal parietal occipital cortex (TPOC), resembles the DMN found in previous anesthetized and awake monkey resting-state fMRI studies [28, 33]. The monkey DMN corresponds well with the MTL sub-network of the human DMN [34, 35]. However, one region missing in our DMN component when compared to humans is a large ventral medial prefrontal cortex (vmPFC) cluster, which is potentially the result of increased susceptibility artifacts.

We observed opposing or inverted responses in the DMN after hpsigma events between the two monkeys. Possible explanations for this result are that it represents normal neurophysiological variability or is a potential side effect of the anesthetic, remifentanil. However, the side-effect explanation is unlikely, since remifentanil is known to have only a negligible effect on neurovascular coupling [36, 37] and only mildly affects the time course and magnitude of neural and vascular responses [38–40]. Further evidence against the side-effect explanation comes from a recent study [41] that found no significant difference between the hippocampal theta rhythm of the anesthetized monkeys analyzed here and the unanesthetized monkey presented in [27].

Future studies that manipulate ripples with concurrent measurements at the level of whole-brain networks can move beyond our correlational results to better explore how ripples might directly modulate endogenous DMN fluctuations. One promising mechanism for how ripples could influence the DMN relates to the high amplitude of hippocampal sharp-waves (an order of magnitude larger than the amplitude of the other neural events), which co-occur with ripples, making them more likely to propagate from the hippocampus [27, 41]. Furthermore, dense hippocampal projections to the retrosplenial and posterior cingulate cortices, regions at the core of the DMN, have been found in rodents [42], macaques [43], and humans [44]. Taken together, these findings suggest that the DMN should be a primary target for propagating activity generated by hippocampal sharp-wave ripples. Neural mass models [45, 46], used with structural connectivity measurements, are ideally suited to theoretically capture the interplay between circuit-level dynamics in the hippocampus and ongoing neocortical BOLD fluctuations. These models could allow researchers to determine whether there are specific types of RSN BOLD signal changes due to hippocampal ripples versus unrelated spontaneous changes.

Our findings are a first step toward capturing the interplay between local neural events in the hippocampus and large-scale RSN dynamics. Notably, the DMN includes neocortical regions important for imagination and episodic memory, allowing for the possibility that hippocampal ripples replay past experience and help the DMN explore potential outcomes of upcoming decisions [2]. Using neural-event-triggered fMRI measurements before and after behavioral training, future studies can potentially characterize how learning modulates neural activity at both DMN and hippocampal circuit levels [3, 46].

EXPERIMENTAL PROCEDURES

Overview

All datasets analyzed were from experiments conducted on two monkeys (E and I) originally published in [27]. All surgical and experimental procedures were approved by the local authorities (Regierungspräsidium, Tübingen Referat 35, Veterinärwesen) and were in full compliance with the guidelines of the European Community (EUVD 86/609/EEC) for the care and use of laboratory animals. Experiments were carried out in two male rhesus monkeys

(*Macaca mulatta*). Neural responses in hippocampal CA1 during resting-state experiments were collected in 50 fMRI experiments/sessions (25 for each monkey) that lasted 10 min each. For details on surgical procedures, see [27]. See Supplemental Experimental Procedures for details on detection of hippocampal ripples and other neural events, maintenance of anesthesia, MRI acquisition, and fMRI preprocessing/statistical analyses.

Resting-State Network Analysis

Spatial ICA, a technique that extracts maximally independent patterns of coherent fMRI activity [47], was applied to each single dataset by means of the GIFT toolbox (http://icatb.sourceforge.net). The estimation of the number of independent components (ICs) was performed using the minimum description length criterion [47]. After reduction of dimensionality by means of principal-component analysis (accounting for at least 99.9% explained variance), ICs were retrieved by means of the FastICA algorithm, with a deflation approach and hyperbolic tangent (*tanh*) nonlinearity [48]. Each fMRI IC consisted of a waveform and a spatial map: the waveform corresponded to the time course of the specific pattern, whereas the associated spatial map expressed the intensity of activity across voxels. To display voxels contributing most strongly to a particular IC and allow intersubject comparison, we scaled the intensity values in each map to Z scores. To extend the ICA analysis from single to multiple datasets, we used the self-organizing group ICA (sogICA) method [48] to sort the ICs extracted from different fMRI datasets and subsequently average them to generate a single IC dataset. SogICA was applied according to a two-stage procedure: first to IC datasets from the same subject for the creation of a representative single-subject IC dataset (within-subject analysis), and then to single-subject IC datasets for the creation of a group-level IC dataset (across-subject analysis). For each sogICA procedure, the IC clusters with relative consistencies $\leq$ 50% or that were spatially correlated at $r > 0.20$ with white matter or CSF patterns (as available in SPM5.0) were excluded from further analyses. The IC clusters obtained at the second level of sogICA were classified as resting-state networks (RSNs).

We performed hierarchical cluster analysis on the entire set of monkey RSNs in common space [49]. To characterize the clustering, we used the spatial correlation as a similarity metric and used the average linkage function. After the creation of the dendrogram, we selected the cutoff value for the graph yielding the maximum number of clusters in both monkeys. This resulted in the definition of single- or two-element clusters. To characterize the reliability of each cluster, we grouped the spatial correlations between the RSNs inside the cluster (intracluster correlations) and those between the RSNs of the cluster and all other RSNs (extracluster correlations). We statistically compared intracluster and extracluster correlations by means of the Mann-Whitney test, thus obtaining a quantitative measure of the cluster reliability. We present RSNs in each subject by averaging the signals across the voxels of the network map (threshold at $Z > 2$). In monkey 1, both the ventral somatomotor and DMN components were present in all 25 datasets. In monkey 2, the ventral somatomotor network component was present in all 25 datasets, while the DMN component was present in 22 datasets. In monkey 2, one dataset did not have any occurrence of gamma events; otherwise there were occurrences of each neural event in every session. Consequently, there were 25 ventral somatomotor and DMN datasets analyzed with monkey 1 and 24 ventral somatomotor and 21 DMN datasets analyzed with monkey 2.

SUPPLEMENTAL INFORMATION

Supplemental Information includes three figures, one table, and Supplemental Experimental Procedures and can be found with this article online at http://dx.doi.org/10.1016/j.cub.2016.01.017.

AUTHOR CONTRIBUTIONS

N.K.L. conceived and designed the experiments. R.K. and G.D. conceived the hypothesis and corresponding analyses. N.K.L. and Y.M. performed the experiments. D.M, M.H.A., and R.H. provided analysis tools. R.K. analyzed the data and wrote the manuscript with input from all of the authors.

ACKNOWLEDGMENTS

This research was supported by the Max Planck Society (N.K.L.), Spanish Research Project PSI2013-42091-P and a European Research Council Advanced grant: DYSTRUCTURE (295129) to G.D., and a Sir Henry Wellcome Fellowship (WT101261MA) to R.K. We apologize to those whose work we have not been able to cite for reasons of space.

Received: November 11, 2015
Revised: December 8, 2015
Accepted: January 5, 2016
Published: February 18, 2016

REFERENCES

1. Raichle, M.E., MacLeod, A.M., Snyder, A.Z., Powers, W.J., Gusnard, D.A., and Shulman, G.L. (2001). A default mode of brain function. Proc. Natl. Acad. Sci. USA *98*, 676–682.
2. Buckner, R.L., Andrews-Hanna, J.R., and Schacter, D.L. (2008). The brain's default network: anatomy, function, and relevance to disease. Ann. N Y Acad. Sci. *1124*, 1–38.
3. Miall, R.C., and Robertson, E.M. (2006). Functional imaging: is the resting brain resting? Curr. Biol. *16*, R998–R1000.
4. Albert, N.B., Robertson, E.M., and Miall, R.C. (2009). The resting human brain and motor learning. Curr. Biol. *19*, 1023–1027.
5. Tambini, A., Ketz, N., and Davachi, L. (2010). Enhanced brain correlations during rest are related to memory for recent experiences. Neuron *65*, 280–290.
6. van Kesteren, M.T., Fernández, G., Norris, D.G., and Hermans, E.J. (2010). Persistent schema-dependent hippocampal-neocortical connectivity during memory encoding and postencoding rest in humans. Proc. Natl. Acad. Sci. USA *107*, 7550–7555.
7. Diekelmann, S., Büchel, C., Born, J., and Rasch, B. (2011). Labile or stable: opposing consequences for memory when reactivated during waking and sleep. Nat. Neurosci. *14*, 381–386.
8. Staresina, B.P., Alink, A., Kriegeskorte, N., and Henson, R.N. (2013). Awake reactivation predicts memory in humans. Proc. Natl. Acad. Sci. USA *110*, 21159–21164.
9. Buzsáki, G., Horváth, Z., Urioste, R., Hetke, J., and Wise, K. (1992). High-frequency network oscillation in the hippocampus. Science *256*, 1025–1027.
10. O'Keefe, J., and Nadel, L. (1978). The Hippocampus as a Cognitive Map (Oxford University Press).
11. Foster, D.J., and Wilson, M.A. (2006). Reverse replay of behavioural sequences in hippocampal place cells during the awake state. Nature *440*, 680–683.
12. Karlsson, M.P., and Frank, L.M. (2009). Awake replay of remote experiences in the hippocampus. Nat. Neurosci. *12*, 913–918.
13. Dupret, D., O'Neill, J., Pleydell-Bouverie, B., and Csicsvari, J. (2010). The reorganization and reactivation of hippocampal maps predict spatial memory performance. Nat. Neurosci. *13*, 995–1002.
14. Buzsáki, G. (1996). The hippocampo-neocortical dialogue. Cereb. Cortex *6*, 81–92.
15. Battaglia, F.P., Benchenane, K., Sirota, A., Pennartz, C.M., and Wiener, S.I. (2011). The hippocampus: hub of brain network communication for memory. Trends Cogn. Sci. *15*, 310–318.
16. Wilson, M.A., and McNaughton, B.L. (1994). Reactivation of hippocampal ensemble memories during sleep. Science *265*, 676–679.
17. Skaggs, W.E., and McNaughton, B.L. (1996). Replay of neuronal firing sequences in rat hippocampus during sleep following spatial experience. Science *271*, 1870–1873.
18. Kudrimoti, H.S., Barnes, C.A., and McNaughton, B.L. (1999). Reactivation of hippocampal cell assemblies: effects of behavioral state, experience, and EEG dynamics. J. Neurosci. *19*, 4090–4101.

19. Nádasdy, Z., Hirase, H., Czurkó, A., Csicsvari, J., and Buzsáki, G. (1999). Replay and time compression of recurring spike sequences in the hippocampus. J. Neurosci. *19*, 9497–9507.

20. O'Neill, J., Senior, T.J., Allen, K., Huxter, J.R., and Csicsvari, J. (2008). Reactivation of experience-dependent cell assembly patterns in the hippocampus. Nat. Neurosci. *11*, 209–215.

21. Ramadan, W., Eschenko, O., and Sara, S.J. (2009). Hippocampal sharp wave/ripples during sleep for consolidation of associative memory. PLoS ONE *4*, e6697.

22. Singer, A.C., Carr, M.F., Karlsson, M.P., and Frank, L.M. (2013). Hippocampal SWR activity predicts correct decisions during the initial learning of an alternation task. Neuron *77*, 1163–1173.

23. Axmacher, N., Elger, C.E., and Fell, J. (2008). Ripples in the medial temporal lobe are relevant for human memory consolidation. Brain *131*, 1806–1817.

24. Girardeau, G., Benchenane, K., Wiener, S.I., Buzsáki, G., and Zugaro, M.B. (2009). Selective suppression of hippocampal ripples impairs spatial memory. Nat. Neurosci. *12*, 1222–1223.

25. Nakashiba, T., Buhl, D.L., McHugh, T.J., and Tonegawa, S. (2009). Hippocampal CA3 output is crucial for ripple-associated reactivation and consolidation of memory. Neuron *62*, 781–787.

26. Ego-Stengel, V., and Wilson, M.A. (2010). Disruption of ripple-associated hippocampal activity during rest impairs spatial learning in the rat. Hippocampus *20*, 1–10.

27. Logothetis, N.K., Eschenko, O., Murayama, Y., Augath, M., Steudel, T., Evrard, H.C., Besserve, M., and Oeltermann, A. (2012). Hippocampal-cortical interaction during periods of subcortical silence. Nature *491*, 547–553.

28. Mantini, D., Gerits, A., Nelissen, K., Durand, J.B., Joly, O., Simone, L., Sawamura, H., Wardak, C., Orban, G.A., Buckner, R.L., and Vanduffel, W. (2011). Default mode of brain function in monkeys. J. Neurosci. *31*, 12954–12962.

29. Mantini, D., Hasson, U., Betti, V., Perrucci, M.G., Romani, G.L., Corbetta, M., Orban, G.A., and Vanduffel, W. (2012). Interspecies activity correlations reveal functional correspondence between monkey and human brain areas. Nat. Methods *9*, 277–282.

30. Deco, G., and Corbetta, M. (2011). The dynamical balance of the brain at rest. Neuroscientist *17*, 107–123.

31. Dragoi, G., and Tonegawa, S. (2011). Preplay of future place cell sequences by hippocampal cellular assemblies. Nature *469*, 397–401.

32. Pfeiffer, B.E., and Foster, D.J. (2013). Hippocampal place-cell sequences depict future paths to remembered goals. Nature *497*, 74–79.

33. Vincent, J.L., Patel, G.H., Fox, M.D., Snyder, A.Z., Baker, J.T., Van Essen, D.C., Zempel, J.M., Snyder, L.H., Corbetta, M., and Raichle, M.E. (2007). Intrinsic functional architecture in the anaesthetized monkey brain. Nature *447*, 83–86.

34. Vincent, J.L., Snyder, A.Z., Fox, M.D., Shannon, B.J., Andrews, J.R., Raichle, M.E., and Buckner, R.L. (2006). Coherent spontaneous activity identifies a hippocampal-parietal memory network. J. Neurophysiol. *96*, 3517–3531.

35. Andrews-Hanna, J.R., Reidler, J.S., Sepulcre, J., Poulin, R., and Buckner, R.L. (2010). Functional-anatomic fractionation of the brain's default network. Neuron *65*, 550–562.

36. Wise, R.G., Rogers, R., Painter, D., Bantick, S., Ploghaus, A., Williams, P., Rapeport, G., and Tracey, I. (2002). Combining fMRI with a pharmacokinetic model to determine which brain areas activated by painful stimulation are specifically modulated by remifentanil. Neuroimage *16*, 999–1014.

37. Pattinson, K.T., Rogers, R., Mayhew, S.D., Tracey, I., and Wise, R.G. (2007). Pharmacological FMRI: measuring opioid effects on the BOLD response to hypercapnia. J. Cereb. Blood Flow Metab. *27*, 414–423.

38. Goense, J.B., and Logothetis, N.K. (2008). Neurophysiology of the BOLD fMRI signal in awake monkeys. Curr. Biol. *18*, 631–640.

39. Logothetis, N.K., Guggenberger, H., Peled, S., and Pauls, J. (1999). Functional imaging of the monkey brain. Nat. Neurosci. *2*, 555–562.

40. Logothetis, N.K., Pauls, J., Augath, M., Trinath, T., and Oeltermann, A. (2001). Neurophysiological investigation of the basis of the fMRI signal. Nature *412*, 150–157.

41. Ramirez-Villegas, J.F., Logothetis, N.K., and Besserve, M. (2015). Diversity of sharp-wave-ripple LFP signatures reveals differentiated brain-wide dynamical events. Proc. Natl. Acad. Sci. USA *112*, E6379–E6387.

42. Jones, B.F., and Witter, M.P. (2007). Cingulate cortex projections to the parahippocampal region and hippocampal formation in the rat. Hippocampus *17*, 957–976.

43. Kobayashi, Y., and Amaral, D.G. (2003). Macaque monkey retrosplenial cortex: II. Cortical afferents. J. Comp. Neurol. *466*, 48–79.

44. Greicius, M.D., Supekar, K., Menon, V., and Dougherty, R.F. (2009). Resting-state functional connectivity reflects structural connectivity in the default mode network. Cereb. Cortex *19*, 72–78.

45. Deco, G., Jirsa, V.K., Robinson, P.A., Breakspear, M., and Friston, K. (2008). The dynamic brain: from spiking neurons to neural masses and cortical fields. PLoS Comput. Biol. *4*, e1000092.

46. Logothetis, N.K. (2015). Neural-Event-Triggered fMRI of large-scale neural networks. Curr. Opin. Neurobiol. *31*, 214–222.

47. Calhoun, V.D., Adali, T., Pearlson, G.D., and Pekar, J.J. (2001). A method for making group inferences from functional MRI data using independent component analysis. Hum. Brain Mapp. *14*, 140–151.

48. Esposito, F., Scarabino, T., Hyvarinen, A., Himberg, J., Formisano, E., Comani, S., Tedeschi, G., Goebel, R., Seifritz, E., and Di Salle, F. (2005). Independent component analysis of fMRI group studies by self-organizing clustering. Neuroimage *25*, 193–205.

49. Mantini, D., Corbetta, M., Romani, G.L., Orban, G.A., and Vanduffel, W. (2013). Evolutionarily novel functional networks in the human brain? J. Neurosci. *33*, 3259–3275.

Current Biology, Volume *26*

Supplemental Information

Hippocampal Sharp-Wave Ripples Influence Selective Activation of the Default Mode Network

Raphael Kaplan, Mohit H. Adhikari, Rikkert Hindriks, Dante Mantini, Yusuke Murayama, Nikos K. Logothetis, and Gustavo Deco

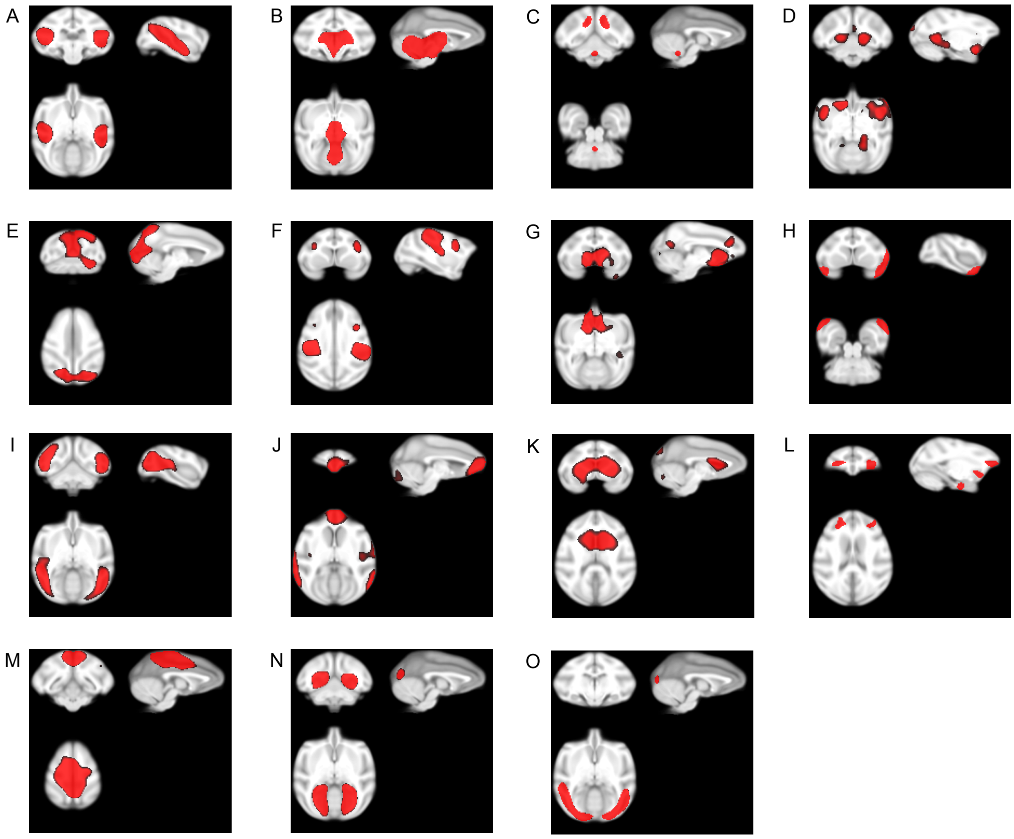

Figure S1, related to Figure 1: Images of all remaining independent components (ICs) identified as resting-state networks present in both monkeys using ICA. A. Occipitotemporal B. Subcortical network including thalamus C. Parieto-cerebellar D. Hippocampal-Prefrontal E. Visual F. Frontoparietal G. Anterior Cingulate H. Temporal pole I. Parieto-occipital J. Frontal pole-temporoparietal K. Basal Ganglia L. Amygdala-Orbitofrontal M. Posterior Cingulate N. Primary Visual O. Middle Visual. Networks shown at slices most representative of the correlation pattern that network identification was based. All images thresholded at Z-score>2 and overlaid on a composite structural from the UWRMAC-DTI271 atlas space.

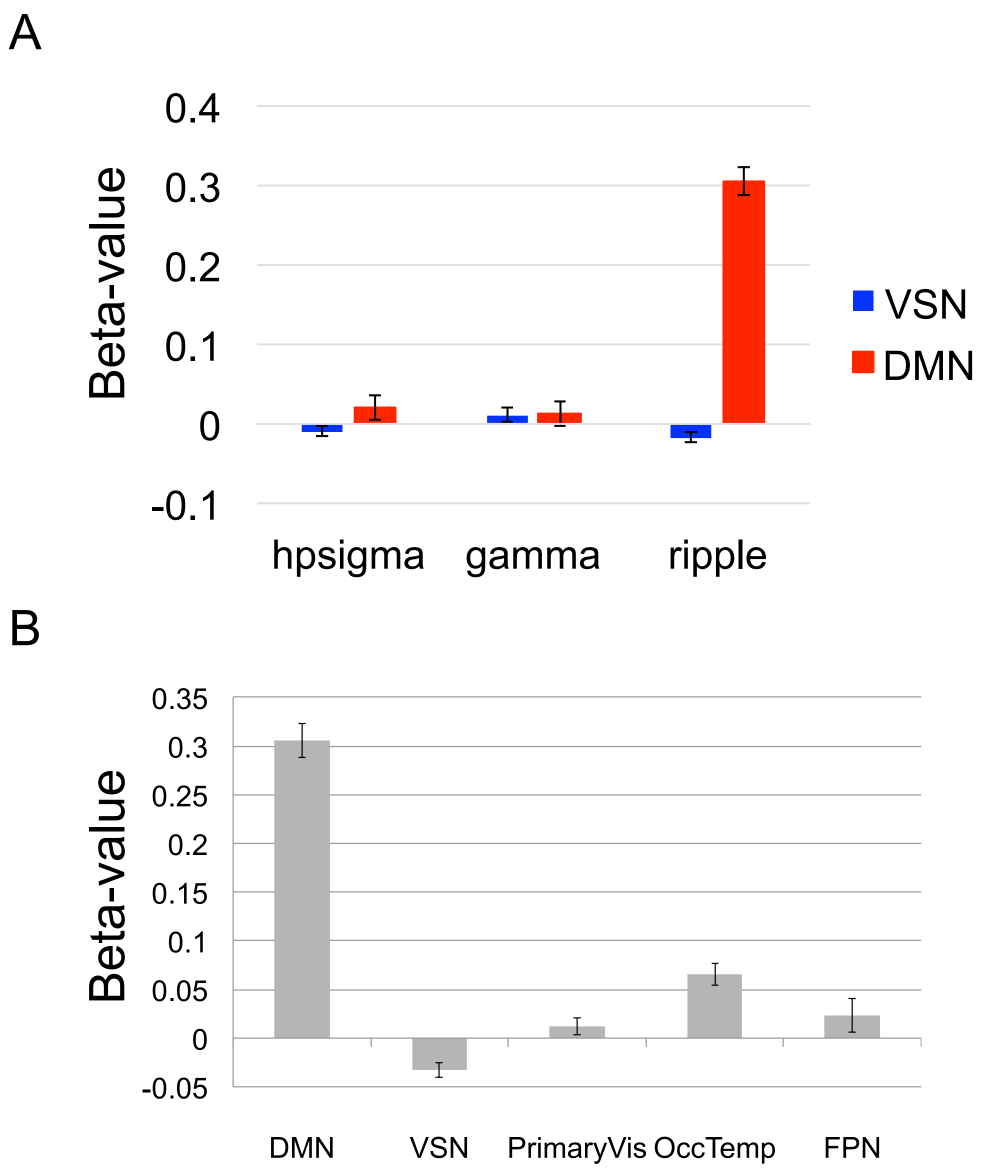

Figure S2, related to Figure 2 and Figure S1: Group Average of fMRI signal changes after ripples in DMN versus other RSNs. A. Beta-values for VSN and DMN after each neural event averaged across both monkeys (mean ± SEM). B. Beta-values after the onset of ripples for DMN, VSN and three other neocortical resting-state networks (Primary Visual, Occipitotemporal, and Frontoparietal) averaged across both monkeys (mean ± SEM). The three other RSNs were chosen, since they were the only neocortical RSNs with ICs present in over half of the 25 datasets in both monkeys. Primary Visual Network ICs (see Figure S1N for anatomical image) were observable in 16 datasets in Monkey 1 and 20 datasets in Monkey 2, Occipitotemporal Network (see Figure S1A for anatomical image) ICs were observable in 24 datasets in Monkey 1 and 14 datasets in Monkey 2, and Frontoparietal Network ICs (see Figure S1F for anatomical image) were observable in 19 datasets in Monkey 1 and 13 datasets in Monkey 2.

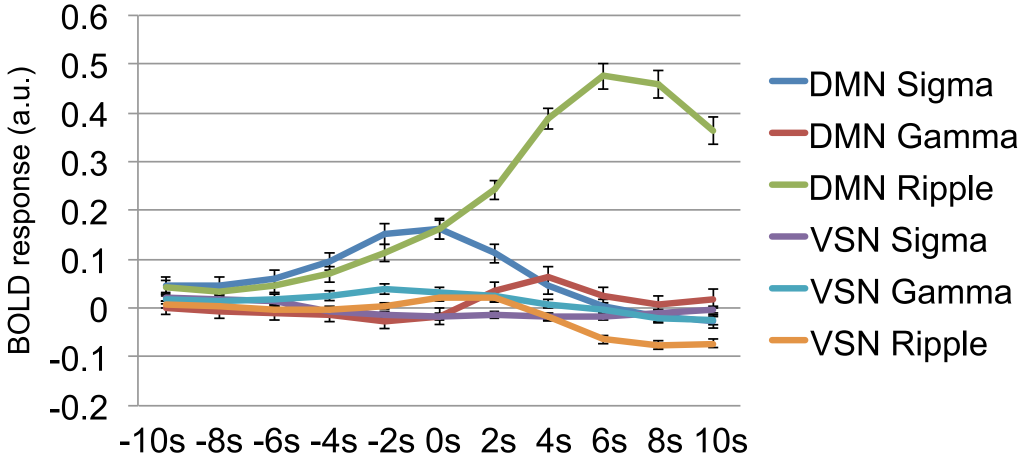

Figure S3, related to Figure 3: Time course of hippocampal neural events in DMN and Ventral Somatomotor Network group average. Evoked responses (signal amplitude presented in arbitrary units) for each network and neural event averaged across both monkeys starting from 5 TRs prior to event onset until 5 TRs after event onset (mean across datasets ± SEM).

Monkey 1	Gamma	Ripple
PCC-Left Hippocampus	.847**	.662**
PCC-Right Hippocampus	.912**	.728**
Monkey 2	Gamma	Ripple
PCC-Left Hippocampus	.267	.528*
PCC-Right Hippocampus	.249	.37

$^{**}p<.001$;$^{*}p<.05$

Table S1, related to Table 1: Correlation between default mode network and hippocampal beta-values.

Pearson correlation coefficient values between individual voxels in left and right hippocampus with a voxel in a key DMN hub, the posterior cingulate cortex (PCC) after gamma and ripple events. Gamma events were used as a control event, since they were not found to significantly influence DMN activity. We found in Monkey 1 that both hippocampal voxels were correlated with the PCC after ripples and gamma events. However in Monkey 2, we only observed a significant correlation between the left hippocampus and DMN after ripples. Beta-values were calculated using the same HRF presented in all other analyses.

Supplemental Experimental Procedures

Ripple and Neural Event Detection

The detection of ripples was made using previously described methodology [S1-3], but with refinements in order to permit classification of events detected by significant power changes across a broad frequency spectrum. Electrode tips were first visually classified into pyramidal cell layer (pl) and stratum radiatum (sr) channels on the basis of their activity patterns (e.g. complex spike characteristics, fast oscillations, SPW-like signal amplitude-deflections. Clear sharp-waves and ripple oscillations could be visibly detected by inspecting the denoised comprehensive or LFP (0.05-250 Hz) signals of each electrode, together with their (80-180 Hz) band-pass-filtered derivative. To detect events, the LFP signal from the pl channels was first filtered between 5 and 200 Hz, rectified, low-pass filtered at 20 Hz with a 4th order Butterworth filter, and finally z-score normalized. Epochs during which the normalized signal exceeded a 3.5 standard deviation threshold were initially considered candidate oscillatory events. After off-line examination of the spectra, the (5-200 Hz) window was reduced to (10-180 Hz), capturing all significant changes occurring during the events in these experiments [S4]. Following the detection of candidate events, spectral analysis using multitaper FFT [S5] was performed on the LFP signal, and the spectra were separated into clusters using the non-negative matrix factorization (NMF) process [S6], an unsupervised feature extraction algorithm permitting the decomposition of multivariate data into a user-defined number of clusters. NMF was used with 10 factorization repetitions. For each spectral cluster, we examined how well a single factor of the NMF results could explain the power spectrum. Factorization quality was quantified using the signal-to-noise (SNR) of the factor, defined as the ration of the sum of the squared power spectrum values to the sum of the squared difference of the spectrum, from its projection on that single factor. Each event was then associated to the factor with the highest SNR, and only “pure” events (i.e. events with almost unimodal spectral distribution) associated to a factor with SNR>3 were selected for further analysis and event-triggered BOLD fMRI. This procedure yielded three robust and distinct clusters in the frequency ranges of 8-22 Hz, 25-75 Hz, and 80-180 Hz, labelled hpsigma, gamma, and ripple ranges respectively. Unlike [S4], which implemented time-course fMRI analysis, where adequate temporal spacing was important, we performed event-related regression analysis, where temporal spacing >1s was not important. Consequently, we did not eliminate neural events occurring within less than one second of each other.

Maintenance of Anesthesia during physiology and fMRI experiments

Experimental recordings were conducted while the animals were under general anesthesia. During the fMRI experiments anesthesia was maintained with remifentanil (0.5-2 μg/kg/min) in combination with a fast-acting paralytic, mivacurium chloride (5-7mg/kg/hr). Anesthesia dosages were determined by measuring stress hormones and were selected to ensure unaffected physiological responses at normal catecholamine concentrations [S4, 7]. Notably, remifentanil is an ultra fast acting μ opioid receptor agonist that has no significant effect on neurovascular activity [S8-9]. Numerous studies [S10-12], including ones using the datasets analyzed in this paper [S4, 13], have shown that remifentanil negligibly affects the magnitude and time course of neural and vascular responses, including the hippocampal LFP [S13]. Additionally, the physiological state of the animal was monitored continuously and maintained tightly within normal limits. Body temperature was maintained at 38-39°C, and end-tidal CO_2 and oxygen saturation were kept constant at 33 mm Hg and over 95%, respectively [S4].

MRI Acquisition

Experiments were conducted in a vertical 4.7 Tesla scanner (Bruker BioSpin, Ettlingen, Germany) with a 40 cm diameter bore. The system has a 50mT/m (180 μs rise time) actively shielded gradient coil (Bruker, BGA26) with an inner diameter of 26 cm. We used a custom-made chair to position the monkey in the magnet and a customized quadrature volume radiofrequency (RF) coil. At the beginning of the experiment, we acquired anatomical images to precisely confirm the electrode position. Coronal sections parallel to the electrode were acquired using a TURBO-RARE sequence with the following parameters: field of view 96 mm x 96 mm, matrix 384x256, in-plane resolution 0.25 mm x 0.375 mm, rare factor 8, effective TE of 60 ms, TR of 3000 ms, BW 38 kHz, 15 slices, slice thickness 0.5 mm and 4 averages. 22 axial slices covering most of the brain were acquired. BOLD activity from these slices was acquired at a TR of 2 seconds with two-shot GE-EPI images (TR/TE=1000/20ms, bandwidth=150 kHz, FA=53o, FOV=96x96mm, matrix=96x96, 2mm slice thickness). In a given experiment, 25 fMRI files (sessions) were acquired containing 300 volumes of 2

seconds duration with spontaneous activity within 4.5 hours. T2-weighted RARE images with the same FOV were obtained using a matrix of 256x256, rare factor 8, effective TE of 60 ms, TR of 5000 ms, BW 42 kHz, and 4 averages. MRI data were analyzed off-line using our own software developed in MATLAB.

fMRI preprocessing

fMRI data preprocessing was performed with the SPM8 software package (www.fil.ion.ucl.ac.uk/spm) running under MATLAB (MathWorks). The first four volumes were discarded to allow for T1 equilibration. Images were coregistered to the anatomical image and spatial normalized to UWRMAC-DTI271 atlas space [S14]. Lastly, data was spatially smoothed with a Gaussian kernel at 5 mm full-width-half-maximum.

Statistical Analyses

Using custom Matlab scripts, we convolved 1117 hpsigma, 823 gamma, and 1720 ripple events in Monkey 1 and 887 hpsigma, 917 gamma, and 911 ripple events in Monkey 2 with the normalized canonical hemodynamic response function (HRF) from SPM8. We subsequently used events as regressors in a standard event-related design. This design gave us a 2x3 within-session repeated measures ANOVA of network (ventral somatomotor and default mode) by event (hpsigma, gamma, and ripple) for each monkey. We then report the corresponding one-sample and paired t-test comparisons after testing for the interaction.

For the time course analysis, we plotted the evoked BOLD response at each TR ranging from 5 TRs before until 5 TRs after the onset of each neural event without fitting to any HRF.

Supplemental References

S1. Buzsáki, G., Horvath, Z., Urioste, R., Hetke, J., Wise, K. (1992). High-frequency network oscillation in the hippocampus. Science *256*, 1025-1027.

S2. Skaggs, W.E., McNaughton, B.L., Permenter, M., Archibeque, M., Vogt, J., Amaral, D.G., and Barnes, C.A. (2007). EEG sharp waves and sparse ensemble unit activity in the macaque hippocampus. J. Neurophysiol. *98*, 898-910.

S3. Sullivan, D., Csicvari, J., Mizuseki, K., Montgomery, S., Diba, K., Buzsaki G. (2011). Relationships between hippocampal sharp waves, ripples, and fast gamma oscillation: influence of dentate and entorhinal cortical activity. J. Neurosci. *31*, 8605-8616.

S4. Logothetis, N.K., Eschenko, O., Murayama, Y., Augath, M., Steudel, T., Evrard, H.C., Besserve, M., and Oeltermann, A. (2012). Hippocampal-cortical interaction during periods of subcortical silence. Nature *491*, 547-553.

S5. Mitra, P.P. and Pesaran, B. (1999). Analysis of dynamic brain imaging data. Biophys. J. *76*, 691-708.

S6. Lee, D.D. and Seung, H.S. (1999) Learning the parts of objects by non-negative matrix factorization. Nature *401*, 788-791.

S7. Canals, S.G., Beyerein, M., Merkle, H., Logothetis, N.K. (2009). Functional MRI Evidence for LTP-Induced Neural Network Reorganization. Curr. Bio. *19*, 398-403.

S8. Wise, R.G., Rogers, R., Painter, D., Bantick, S., Ploghaus, A., Williams, P., Rapeport, G., Tracey, I. (2002). Combining fMRI with a pharmacokinetic model to determine which brain areas activated by painful stimulation are specifically modulated by remifentanil. Neuroimage *16*, 999-1014.

S9. Pattinson, K.T., Rogers, R. Mayhew, S.D., Tracey, I., Wise, R.G. (2007). Pharmacological FMRI: measuring opioid effects on the BOLD response to hypercapnia. J. Cereb. Blood Flow. Metab. *27*, 414-423.

S10. Goense, J.B. and Logothetis, N.K. (2008). Neurophysiology of the BOLD fMRI signal in awake monkeys. Curr. Bio. *18*, 631-640.

S11. Logothetis, N.K., Guggenberger, H., Peled, S., Pauls, J. (1999). Functional imaging of the monkey brain. Nat. Neurosci. *2*, 555-562.

S12. Logothetis, N.K., Pauls, J., Augath, M., Trinath, T., Oeltermann, A. (2001) Neurophysiological investigation of the basis of the fMRI signal. Nature *412*, 150-7.

S13. Ramirez-Villegas, J.F., Logothetis, N.K., Besserve, M. (2015). Diversity of sharp-wave-ripple LFP signatures reveals differentiated brain-wide dynamical events. Proc. Natl. Acad. Sci. U.S.A. *112*, E6379-6387.

S14. Adluru, N., Zhang, H., Fox, A.S., Shelton, S.E., Ennis, C.M., Bartosic, A.M., Oler, J.A., Tromp, D.P.M., Zakszewski, E., Gee, J.C., Kalin, N.H., and Alexander, A.L. (2012). A Diffusion Tensor Brain Template for Rhesus Macaques. Neuroimage *59*, 306-318
